# The miRNA Profile of Human Pancreatic Islets and Beta-Cells and Relationship to Type 2 Diabetes Pathogenesis

**DOI:** 10.1371/journal.pone.0055272

**Published:** 2013-01-25

**Authors:** Martijn van de Bunt, Kyle J. Gaulton, Leopold Parts, Ignasi Moran, Paul R. Johnson, Cecilia M. Lindgren, Jorge Ferrer, Anna L. Gloyn, Mark I. McCarthy

**Affiliations:** 1 Oxford Centre for Diabetes, Endocrinology & Metabolism, University of Oxford, Oxford, United Kingdom; 2 Wellcome Trust Centre for Human Genetics, University of Oxford, Oxford, United Kingdom; 3 Wellcome Trust Sanger Institute, Hinxton, United Kingdom; 4 Donnelly Centre for Cellular and Biomolecular Research, Toronto, Canada; 5 Genomic Programming of Beta-cells Laboratory, Institut d'Investigacions August Pi I Sunyer (IDIBAPS), Barcelona, Spain; 6 Nuffield Department of Surgery, University of Oxford, Oxford, United Kingdom; 7 Oxford NIHR Biomedical Research Centre, Churchill Hospital, Oxford, United Kingdom; Colorado State University, United States of America

## Abstract

Recent advances in the understanding of the genetics of type 2 diabetes (T2D) susceptibility have focused attention on the regulation of transcriptional activity within the pancreatic beta-cell. MicroRNAs (miRNAs) represent an important component of regulatory control, and have proven roles in the development of human disease and control of glucose homeostasis. We set out to establish the miRNA profile of human pancreatic islets and of enriched beta-cell populations, and to explore their potential involvement in T2D susceptibility. We used Illumina small RNA sequencing to profile the miRNA fraction in three preparations each of primary human islets and of enriched beta-cells generated by fluorescence-activated cell sorting. In total, 366 miRNAs were found to be expressed (i.e. >100 cumulative reads) in islets and 346 in beta-cells; of the total of 384 unique miRNAs, 328 were shared. A comparison of the islet-cell miRNA profile with those of 15 other human tissues identified 40 miRNAs predominantly expressed (i.e. >50% of all reads seen across the tissues) in islets. Several highly-expressed islet miRNAs, such as miR-375, have established roles in the regulation of islet function, but others (e.g. miR-27b-3p, miR-192-5p) have not previously been described in the context of islet biology. As a first step towards exploring the role of islet-expressed miRNAs and their predicted mRNA targets in T2D pathogenesis, we looked at published T2D association signals across these sites. We found evidence that predicted mRNA targets of islet-expressed miRNAs were globally enriched for signals of T2D association (p-values <0.01, q-values <0.1). At six loci with genome-wide evidence for T2D association (*AP3S2*, *KCNK16*, *NOTCH2*, *SCL30A8*, *VPS26A*, and *WFS1*) predicted mRNA target sites for islet-expressed miRNAs overlapped potentially causal variants. In conclusion, we have described the miRNA profile of human islets and beta-cells and provide evidence linking islet miRNAs to T2D pathogenesis.

## Introduction

The overwhelming majority of the disease-associated variation identified by genome-wide association studies (GWAS) maps to the non-protein-coding genome. Efforts to unlock the functional impact of these variants therefore rely on an understanding of the processes involved in the regulation of transcription, most particularly those which are active in the cells and tissues implicated in disease pathogenesis [1], [2].

miRNAs, short (∼22 nucleotides) non-coding RNAs, are thought to play a key role in the regulation of cellular function through effects on mRNA destabilisation and/or translational repression [3], [4]. Altered miRNA function has been implicated in the pathogenesis of a growing number of diseases, including Tourette's syndrome and a variety of cancers [5], [6]. There is also substantial evidence linking miRNAs to the regulation of glucose homeostasis. For example, miR-375 has been reproducibly shown to be involved in the regulation of glucose-stimulated insulin secretion in the murine insulin-secreting cell-line MIN6 [7], [8] and other miRNAs (let-7, miR-103 and -107) influence insulin sensitivity in rodents [9], [10].

Many of the variants robustly associated with type 2 diabetes (T2D) in GWAS exert their diabetogenic effect via a primary reduction in insulin secretion, placing the pancreatic islet, and the insulin-secreting beta-cell in particular, center-stage in terms of T2D pathogenesis [11]. Given significant differences in islet physiology between rodents and humans [12], and with few suitable human beta-cell lines available [13], the genomic characterisation of primary human islet preparations provides an important opportunity to develop the functional annotations that can support biological inference at T2D association signals.

In this study, we set out to define the miRNA profile of primary human islets and enriched human beta-cell preparations, and to relate these findings to patterns of T2D predisposition.

## Results

### The miRNA profile of human pancreatic islets and beta-cells

Human islets were acquired from six donors with appropriate ethical consent. For three of these samples the islets were subjected to fluorescence-activated cell sorting (FACS) to select for beta-cells. The purity of the beta-cells following FACS ranged between 90–95%, and we refer to these as “enriched beta-cell” preparations. All six samples (3 islet and 3 enriched beta-cells; see Table S1 for clinical characteristics) were sequenced using 50 base pair reads on either Illumina GAIIx or Hiseq2000 platforms. After quality control, a total of 83 million reads mapping to small RNA species were obtained across the three islet samples (648,000, 14 million, 68 million per sample), and 68 million across the enriched beta-cell samples (3 million, 38 million, 26 million per sample). The differences in read-depths reflect improvements in sequencing technology over the period of data collection.

Almost all reads (92%) mapped to mature miRNA sequences annotated in miRBase v18 [14], the remainder being snoRNAs (2.4%) and other non-coding species in Ensembl v63 [15]. As the number of annotated human miRNAs is still expanding, we used the miRDeep2 package – a probabilistic method for discovering miRNAs from small RNA sequencing data using the predicted secondary structure of potential miRNA precursors [16] – to uncover novel, unannotated miRNAs in our sequencing data, but there were no instances of high confidence predictions of novel miRNAs.

**Figure 1:**
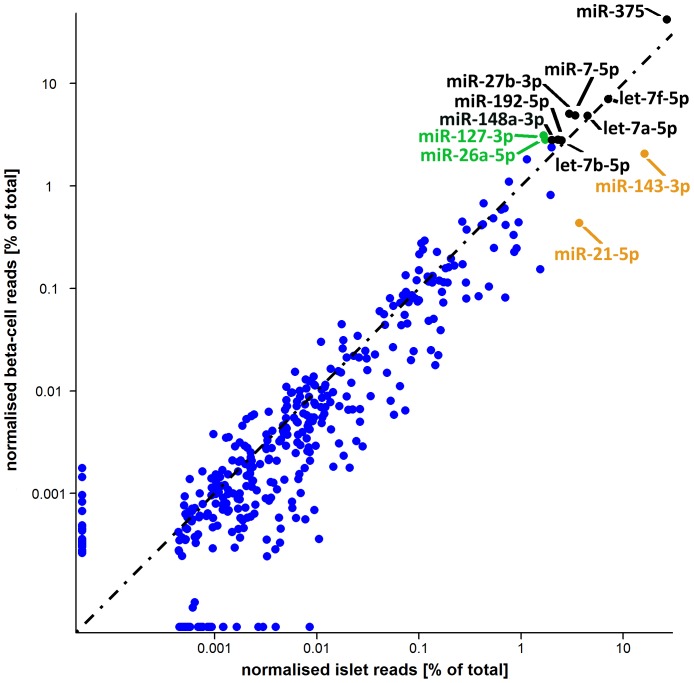
Islet and beta-cell miRNA profiles. The top 10 most abundant miRNAs in islets and beta-cells are annotated, with colors denoting those shared between the two top 10s (black), in islet top 10 only (orange), and in beta-cell top 10 only (green). There was good correlation between the two profiles (r^2^ = 0.78). A black dashed line represents equality.

In islets, 366 miRNAs were expressed above background levels (i.e. more than 100 combined reads across the three samples), and 346 in enriched beta-cells (Figure 1; Table S2). In total, 384 unique miRNAs were identified, of which 85% (n = 328) were shared between islets and beta-cells (Table S2). The expression profile was of medium complexity with on average 38 miRNAs responsible for 90% of the total miRNA aligned reads in islets, and 21 in enriched beta-cells. There was good reproducibility across the three samples of each type (Spearman rho range 0.61–0.96), with all but one expressed miRNA present in at least two samples of each type.

Several of the most highly expressed miRNAs in human islets (including miR-375, miR-7-5p, miR-148a-3p, miR-26a-5p and miR-127-3p) have been previously described in rodent or human islets [7], [17]–[20]. The expression in islets of others (e.g. miR-27b-3p, miR-192-5p) is novel. Other islet-expressed miRNAs such as miR-143-3p and let-7 family members have been implicated in glucose homeostasis, but primarily in the context of insulin action rather than insulin secretion [10], [21], [22].

Comparison of the miRNA expression profiles of islets and enriched beta-cells revealed a strong correlation between the two (Figure 1), but highlighted some interesting differences. For example, amongst the most highly expressed miRNAs, miR-375 shows substantially higher abundance in enriched beta-cells as compared to islets (42% vs 27%) whilst the opposite is true for miR-143-3p (2% vs 16%). On the basis that an average islet contains ∼50% beta-cells [23], and, given an estimated 90% purity of the FACS-enriched beta-cell preparations, the ∼1.5 fold enrichment of miR-375 in beta-cells compared to islets indicates that, in human as in rodents [7], this miRNA is predominantly expressed in beta-cells. On the same grounds, these data suggest that miR-143-3p is mostly expressed outside pancreatic beta-cells.

### Tissue specificity of miRNA expression

We compared the miRNA expression profiles in islets against equivalent, publicly available, next-generation sequencing-derived miRNA expression data from other human tissues, including B-cells [24], liver [25], pigment cells [26], pooled thymocytes, bone marrow, CD34+ progenitor cells [27], skin [28], lung, kidney, skeletal muscle, heart, whole pancreas, frontal orbital gyrus, spleen, liver tissue [29], and adipose tissue [30]. Raw reads were downloaded, realigned and normalized in parallel with our islet and beta-cell samples (see Methods). From these data, we generated a tissue specificity score (see Methods), defined as the expression of a miRNA in the reference tissue divided by the sum of its expression in all tissues, and considered a value >0.5 to indicate tissue-specificity.

**Figure 2:**
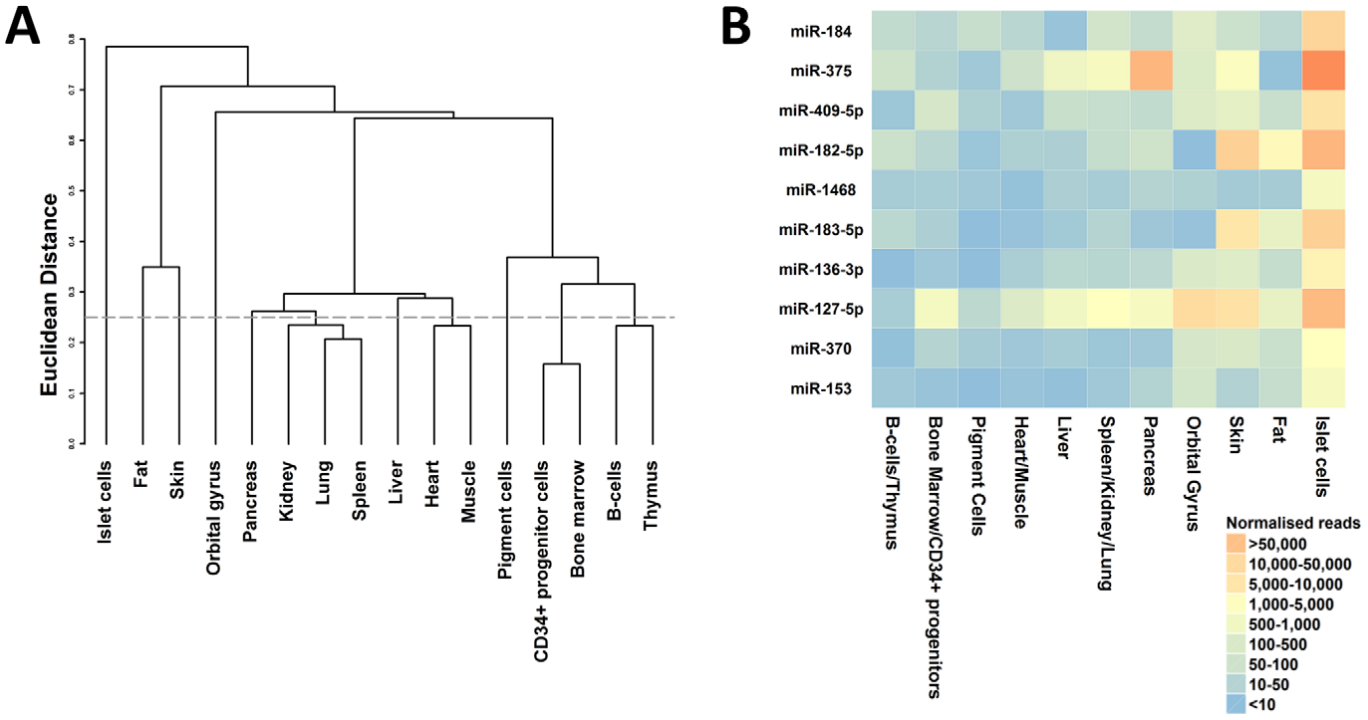
Comparison of miRNA profiles across tissues. The left panel (A) shows the single-linkage hierarchical clustering of inter-tissue profile correlations. In the right panel (B) the top 10 most tissue specific islet miRNAs are displayed in descending order. The colors indicate the normalized expression levels of these miRNAs across the different profiles used in the analysis.

By this definition (which indicated that half or more of all observed reads for that miRNA were derived from islets) 40 of the 366 islet miRNAs were islet-specific (Table S3). Equivalent specificity analyses using other tissues as reference found a mean of only 10 enriched miRNAs per tissue (ranging from 1 [in spleen, kidney and lung] to 31 [orbital gyrus]; Table S3). Inevitably, these comparisons are influenced by the panel of tissues for which data were available, but they suggest that the miRNA profile of human islets is relatively distinct (Figure 2A). By permuting the data, we estimated that the false-discovery rate associated with an assertion of tissue specificity for a given miRNA was approximately 8%.

Few of the 10 most islet-specific miRNAs (Figure 2B; all with specificity scores >0.8) have previously been implicated in islet function. For miR-184, miR-182-5p and miR-127-3p, there is published evidence for a role in insulin biosynthesis and secretion, though for miR-184 and miR-127-3p this is restricted to a correlation between islet expression levels and glucose-stimulated insulin secretion [17], [18]. For other miRNA transcripts, such as miR-409-5p and miR-183-5p, the high degree of islet-specificity may point to novel roles in the development and maintenance of islet cellular phenotype.

Primary miRNA sequences tend to cluster together in genomic intervals spanning <10 kb, and it is thought that members of these clusters are transcribed together [30], [31]. We found examples of such clustering amongst the miRNAs showing the greatest absolute abundance in islets, as well as those that were most islet-specific. For example, mir-182 and mir-183 (both amongst the most islet-specific transcripts) originate from the same cluster on chromosome 7q32.2, whilst mir-136 and mir-127 map together on chromosome 14q32.2. Across samples, expression levels of miRNAs within these clusters are highly correlated both in islets and enriched beta-cells (r^2^>0.6).

### The role of islet-expressed miRNAs in T2D predisposition

To explore the role in T2D predisposition played by genetic variants that influence miRNA function and/or expression, we analysed genome-wide association data from 8,130 T2D cases and 38,987 controls available through the Diabetes Genetics Replication and Meta-analysis (DIAGRAM) consortium [11].

We first looked for evidence that T2D-associated variants mapped to sequences encoding miRNAs themselves. Of the ∼2.5 million variants for which directly-typed or HapMap-imputed T2D-association p-values were available, seven overlapped precursor miRNA transcripts for islet-expressed mature miRNAs (there were a total of 364 of these in miRBase v18 corresponding to the 366 mature miRNAs we document in islets).

Next, we asked whether the predicted targets of islet-expressed miRNAs were enriched for evidence of association with T2D. To determine whether results were robust to different algorithms for predicting these target sites, we used three different prediction algorithms (TargetScan, miRanda and miRDB) [32]–[34]. As two of these algorithms (TargetScan and miRanda) provide genomic coordinates for the target sites they predict, we were able, for these, to consider both the target genes and these specific target sites. Looking at the predicted target sites of those 366 islet miRNAs (a total of 39,103 and 749,873 such target sites were predicted by TargetScan and miRanda respectively), we identified 6,496 variants overlapping the association data. The relatively low density of common variants observed in both islet miRNA and miRNA target sequence, is consistent with the constraints imposed by negative selection [19], [35], [36]. Of the 6,503 variants overlapping miRNA transcripts and predicted miRNA target sites, one variant, rs3802177 at the *SLC30A8* locus (p-value = 1.45×10^−8^) showed genome-wide significant association for T2D in the DIAGRAM meta-analysis data.

We also sought overlap with miRNA sequence and/or predicted targets within the set of 58 loci for which there was consistent evidence for genome-wide significant associations with T2D (as of July 2012 [11], [37], [38]). As the causal variant at most T2D-association loci is not known, we defined a broad set of 1,403 variants comprising the lead SNPs at the 58 loci plus all good proxies (LD; r^2^>0.8 in CEU 1000 Genomes pilot data) which was likely to include the alleles causal for these associations. We found overlap of target sites (all predicted by miRanda) at ten of these variants, mapping to six independent loci (*AP3S2, KCNK16, NOTCH2, SLC30A8, VPS26A*, and *WFS1*).

**Figure 3:**
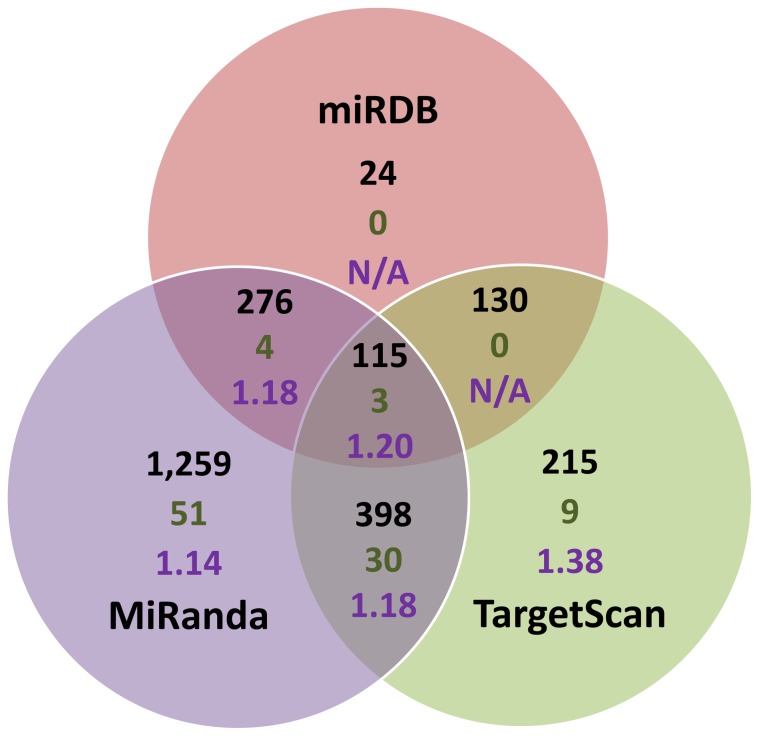
Results for the T2D association signal enrichment analysis in miRNA target gene sets. The Venn diagram represents the sets of miRNA target genes for each miRNA as predicted by miRDB, miRanda and TargetScan alone, or overlap of these methods. Annotated are the median number of genes in each set (black text), the number of significantly (p-value <0.001, q-value <0.1) enriched gene sets (green text), and the median enrichment of the significantly enriched gene sets (purple text).

Finally, we implemented an approach where we sought evidence for enrichment of T2D association signals across sets of islet-expressed genes predicted to be targeted by an individual islet-expressed miRNA. As we used three different target prediction algorithms, with often differing predicted target genes for individual miRNAs, we looked at the degree of enrichment of signal in the target genes predicted by each method alone, or by the overlap (Figure 3). To test for enrichment within the predicted transcriptional targets of each miRNA, we employed MAGENTA [39], a tool that uses meta-analysis summary statistics to determine enrichment for trait association across a given gene set while correcting for differences in transcript size, number of variants, and patterns of LD. We found significant enrichment (p-values <0.01, q-value <0.1) across islet miRNA targeted gene sets with nearly all of the (combinations of) prediction algorithms used, with significant enrichment (miR-17-5p, p-value = 0.004, q-value = 0.09; miR-93-5p, p-value = 0.005, q-value = 0.09; miR-20a-5p, p-value = 0.009, q-value = 0.09) even in the set of targets predicted by all three different algorithms (Figure 3, Table S4).

## Discussion

Using next-generation sequencing, we have established the first catalog of miRNAs in human pancreatic islets and beta-cells, and explored the overlap between these miRNAs and T2D genetic susceptibility. Our catalog not only serves as a valuable resource for those interested in the roles of specific miRNAs in normal islet physiology and beta-cell function, it also provides a reference for the study of miRNA mediated abnormalities in islets from type 2 diabetic donors.

The abundance of miR-375 in the miRNA profile provides valuable support for a critical role in human pancreatic beta-cells, mirroring the well-established role in rodent islet biology. miR-375 null mice are hyperglycaemic and exhibit reduced beta-cell mass [40]. In a clonal rodent beta-cell line (MIN6), knockdown or over-expression of this miRNA influences glucose-stimulated insulin secretion [7]. Furthermore, knockdown of miR-375 in obese ob/ob mice results in a more profound effect on glycaemia leading to a severe diabetic phenotype in these mice [40]. Our study establishes that miR-375 is also abundantly expressed in human islets and warrants further studies to define the contribution of miR-375 to the pathogenesis of T2D.

Most of the other abundant miRNAs show comparable levels of expression between islets and beta-cells, and the islet-cell profiles are relatively distinct from most of the other tissues studied. Our data adds support for an islet-enriched expression pattern of six miRNAs previously highlighted by microarray studies (miR-184, miR-183-5p, miR-7-5p, miR-127-3p, miR-375, and miR-493-5p) [17], [20]. However, for many of the miRNAs that are most abundant and/or enriched in human islets, there is little, if any, existing data concerning their effects on islet development or function. For example, these data point to the need to further functional investigation of the let-7 family of miRNAs. Two recent studies have demonstrated that manipulation of let-7 levels in mice, by overexpression or knockdown, alters insulin sensitivity [10], [22]. One of these studies however also showed reduced insulin secretion during an intraperitoneal glucose tolerance test in the let-7 overexpressing mice, hinting at a role for let-7 in glucose-stimulated insulin secretion, but did not characterize the effects of altered let-7 expression on pancreatic islet physiology [22].

When we investigated the overlap between the islet miRNAs and published T2D genome-wide association data, we found evidence suggestive of a role for miRNAs in diabetes pathogenesis. We identified both global enrichment of T2D association signals within islet-expressed miRNA target genes, and overlap of potentially causal T2D-associated variants with predicted target sites for islet-expressed miRNAs.

The strongest signal of overlap we identified was for a variant (rs3802177) within the 3′ UTR of the *SLC30A8* gene, which maps to miRanda predicted target sites for six islet-expressed miRNAs (miR-363-3p, miR-25-3p, miR-32-5p, miR-92a-3p, miR-33a-5p, and miR-33b-5p) and reaches genome wide significance in T2D-association studies [11]. Whilst, in the DIAGRAM meta-analysis, rs3802177 is the variant with the strongest association with T2D [11], it is also in complete linkage disequilibrium with a non-synonymous coding variant in *SLC30A8* (rs13266634), which is generally considered to be the causal allele [41], [42]. Further functional studies will be required to establish whether rs3802177 could be contributing to perturbation of SLC30A8 expression and/or function.

These data are based on the common variant associations hitherto accessible to interrogation by GWAS. Extension of risk variant discovery efforts through next-generation sequencing to include low frequency and rare alleles will allow a more complete evaluation of the role of miRNAs and their targets with respect to pancreatic islet development and/or function and the pathogenesis of T2D.

## Materials and Methods

### Samples

Human islets were obtained (with research consent) from the Oxford DRWF Human Islet Isolation Facility (n = 4) and through existing collaborations with Barcelona (n = 2) from deceased donors of European descent (clinical characteristics of the donors can be found in Table S1). High islet purity was established both by dithizone labeling, as well as through comparative quantification by qRT-PCR of endocrine (insulin [*INS*], glucagon [*GCG*], somatostatin [*SST*]) and exocrine (pancreatic lipase [*PNLIP*], pancreatic amylase [*AMY2A*] and chymotrypsin C [*CTRC*]) markers.

The beta-cell enriched preparation was collected through fluorescence-activated cell sorting of human islet preparations using a previously described method [43], [44]. RNA was extracted from all samples using TRI reagent (Applied Biosystems, Warrington, UK).

### Library preparation and sequencing

Only samples with a total RNA yield in excess of 1ug and a quality score (RIN) >7 were selected for library preparation. Libraries were prepared at the High-Throughput Genomics group (Wellcome Trust Centre for Human Genetics, University of Oxford, Oxford, UK) using Illumina v1.5 (2 islets and 1 beta-cell sample) or TruSeq v1 (2 beta-cell and 1 islet sample) small library preparation protocols, and sequenced using 50 base reads on Illumina GAIIx and HiSeq2000 platforms respectively.

### Raw sequence processing

Raw data was obtained in Fastq format, and pre-processed to remove residual 3′-adaptor sequences using the fastx_clipper function from the FASTX-toolkit (http://hannonlab.cshl.edu/fastx_toolkit/index.html). Sequences less than 16 bases after adaptor stripping, and reads containing primarily N bases, were removed. The remaining read sequences should correspond to short RNAs. Length histograms showed enrichment of a peak around 22 bases, which corresponds to the expected size of miRNAs.

### Sequence mapping and quantification

As ambiguities in read mapping, and frequent contamination by large numbers of adaptor dimers pose significant challenges to the accurate quantification of small RNAs, we used a three step alignment approach. Reads were aligned using novoalign v. 2.07.11 (http://www.novocraft.com, parameters -h 60 60 -t30 -s -m -l 16 -R 0 -r A 30), which is aware of sequence ambiguities, to three consecutive references:

1. Contaminating sequences of adaptors, linkers, adaptor-linker, and adaptor-tag combinations.
2. Full-length miRBase v17 hairpin sequences, combined with other known and predicted human small RNA sequences present in Ensembl v63 [14], [15].
3. Human reference genome (NCBI build 37).

Reads mapping to contaminating sequences were excluded. The remaining reads were prioritised in order miRNA > ncRNA > genome, and alignments with the smallest edit distance were selected. miRNA hairpin aligning reads were further split out into mature miRNAs, star miRNAs and those reads mapping only to the hairpin by using the respective coordinates on the hairpin from miRBase v17 extended by 3 bases either side of the mature and star sequences.

Finally, expression levels of each ncRNA were quantified by counting the number of reads aligning to it. In case of multiple mapping, where a read mapped between *k* alternative sequences in one reference, *1/k* was added to the count of each. Those RNA sequences observed less than 100 times across all samples were excluded. This cut-off was chosen as the reproducibility across samples dropped when going lower, and means that per sample >95% of all mapped sequences are included in the analysis.

### Normalization

Data was normalized to be able to compare read counts between samples, which all differ in read depth. An inflation factor *i* was calculated for each library *l* using a method proposed by Anders and Huber [45]. This was determined as the median inflation factor across all genes *g* using the following formula: *il*  =  median_g_(n*_gl_*/GM*_l_*(n*_gl_*)), where n*_gl_* is the read count each gene *g* in that library *l*, and GM is the geometric mean. Next, the data was log2-transformed (log_2_(*n_gl_*/*il*)) to to account for the heteroskedastic distribution of the data. These values were used in all subsequent analyses.

### Tissue specific analysis

Publicly available human small RNA sequencing data using Illumina technology from B-cells [24], liver [25], pigment cells [26], pooled thymocytes, bone marrow, CD34+ progenitor cells [27], skin [28], lung, kidney, skeletal muscle, heart, pancreas, frontal orbital gyrus, spleen, and liver tissue [29], as well as to data from adipose tissue available through collaboration with the MuTHER consortium [30], were downloaded, realigned and normalized together with the islet and beta-cell samples. To minimalize background, only those miRNAs observed at least 1000 times across the normalized values (n = 367) were taken forward for tissue specific analysis.

Subsequently we calculated a tissue specificity score, defined as the expression of a miRNA in a tissue divided by the sum of its expression in all tissues, for each miRNA in each tissue. The resulting fractional expression is an indicator of what proportion of the expression of that miRNA is attributable to a given tissue. As this approach would be limited in a set of tissues containing very similar profiles, we combined the results from the islets and the beta-cells and calculated a correlation matrix between the different profiles and subjected this to single-linkage hierarchical clustering in R version 2.14.0 using *hclust* [46]. To create profiles as unrelated as possible, all tissues with a Euclidian distance <0.25 were averaged along their branch. For each of the remaining 11 profiles, tissue specific scores were calculated by dividing the normalized reads for a miRNA in a tissue by all reads in all tissue for that miRNA. The resulting score thus represented the fraction of miRNA reads explained by that tissue. Tissue-specificity was defined by a score >0.5, which, using a permutation-based approach comprising 1 million permutations of the observed data, amounts to a false-discovery rate of 8%.

### Involvement of miRNAs in T2D association

For all reported T2D loci, variants in strong LD (R^2^>0.8) in 1000 Genomes pilot CEU data were combined with the reported T2D most associated variants using custom scripts. Genomic locations of miRNAs were downloaded from miRBase v18 (http://www.mirbase.org) [14], Targetscan 6.2 [32] conserved predictions from http://www.targetscan.org, miRanda predicted targets with good mirSVR scores (release August 2010) [33] from http://www.microrna.org/microrna/getDownloads.do and miRDB (version 4) [34] predicted target genes from http://mirdb.org/miRDB/download.html.

For the overlap with T2D associated variants, genomic coordinates were, where appropriate, converted to hg18 using the UCSC liftOver tool with -*minMatch = 1*. For the enrichment analysis we determined the intersection between predicted islet-expressed target genes of all three algorithms for each miRNA using custom Perl scripts. MAGENTA [39] was run using pre-defined settings using 100000 permutations for the p-value calculation, and the 75*^th^* percentile cut-off p-values for establishing enrichment. To correct for multiple testing q-values, which represent the expected proportion of false positives incurred when calling that gene set significant, were calculated using the *qvalue* command from the package “qvalue” in R version 2.14.0 [46] with the setting “robust = TRUE”. Those gene sets with p-values <0.01 and q-values <0.1 were deemed significant.

### Accession numbers

Datasets have been deposited in Gene Expression Omnibus GEO (In process).

## Supporting Information

Table S1 Clinical characteristics of human islet donors. N/A denotes information not available.
(DOCX)

Table S2 Normalized average read counts for all 385 miRNAs present in human islets and beta-cells. Each row denotes an individual miRNA present above background levels (>100 observed reads). The first column has the miRNA identifiers according to miRBase 18. The entries in the two columns are the normalised average read counts for, respectively, human islets and beta-cells. 0 denotes the expression of the miRNA was below background levels (<100 total reads observed).
(DOCX)

Table S3 miRNA level comparison between tissues. Each row represents a different miRNA observed for at least 1000 reads across all tissues. The first column has the miRNA identifiers according to miRBase 18. Each other column represents a distinct (set of) tissues used for the comparison. The entries in the columns denote the tissue specificity score as described in the Methods.
(DOCX)

Table S4 Results for the significantly enriched predicted target gene sets for islet-expressed miRNAs from the MAGENTA analysis with the 75 percentile cut-off used to determine significance.
(DOCX)
