# Supplementary material for: The miRNA Profile of Human Pancreatic Islets and Beta-Cells and Relationship to Type 2 Diabetes Pathogenesis
Source: PLoS One. 2013 Jan 25;8(1):e55272. doi: 10.1371/journal.pone.0055272 (PMC3555946; doi:10.1371/journal.pone.0055272)
Supplement: Table S1 — Clinical characteristics of human islet donors. N/A denotes information not available. (DOCX) [file pone.0055272.s001.docx]

|  | **Isl1** | **Isl2** | **Isl3** | **Beta1** | **Beta2** | **Beta3** |
| --- | --- | --- | --- | --- | --- | --- |
| **Sex** | Male | Female | Female | Male | Female | Female |
| **Age (years)** | 59 | 57 | 32 | 41 | 51 | 59 |
| **Cause of death** | Ischemic brain injury | Ischemic brain injury | Trauma | Meningioma | N/A | N/A |
| **BMI (kg/m2)** | 24 | 36 | 27 | 29 | 31 | 29 |
| **Pre-mortem diagnosis of diabetes** | No | No | No | No | No | No |
| **Cold ischemia (hours)** | 6.4 | 8.5 | 8 | 8.5 | 5 | 7.5 |
| **Islet purity (%)** | 94 | 75 | 80 | 80 | 90 | 70 |
