# Supplementary material for: The miRNA Profile of Human Pancreatic Islets and Beta-Cells and Relationship to Type 2 Diabetes Pathogenesis
Source: PLoS One. 2013 Jan 25;8(1):e55272. doi: 10.1371/journal.pone.0055272 (PMC3555946; doi:10.1371/journal.pone.0055272)
Supplement: Table S2 — Normalized average read counts for all 385 miRNAs present in human islets and beta-cells. Each row denotes an individual miRNA present above background levels (>100 observed reads). The first column has the miRNA identifiers according to miRBase 18. The entries in the two columns are the normalised average read counts for, respectively, human islets and beta-cells. 0 denotes the expression of the miRNA was below background levels (<100 total reads observed). (DOCX) [file pone.0055272.s002.docx]

| **miRNA** | **Normalized Islet Average** | **Normalized Beta-cell Average** |
| --- | --- | --- |
| hsa-miR-375 | 2,046,518 | 5,804,575 |
| hsa-miR-143-3p | 1,233,087 | 284,207 |
| hsa-let-7f-5p | 544,989 | 966,619 |
| hsa-let-7a-5p | 343,033 | 668,000 |
| hsa-miR-21-5p | 284,834 | 60,031 |
| hsa-miR-7-5p | 259,457 | 670,521 |
| hsa-miR-27b-3p | 226,272 | 698,396 |
| hsa-miR-192-5p | 190,726 | 383,622 |
| hsa-miR-148a-3p | 176,002 | 389,911 |
| hsa-let-7b-5p | 153,729 | 386,363 |
| hsa-miR-182-5p | 152,455 | 327,398 |
| hsa-miR-30d-5p | 149,301 | 111,933 |
| hsa-miR-26a-5p | 132,411 | 389,655 |
| hsa-miR-127-5p | 126,729 | 427,356 |
| hsa-miR-30a-5p | 117,912 | 21,116 |
| hsa-miR-22-3p | 87,376 | 250,404 |
| hsa-miR-200c-3p | 72,125 | 60,837 |
| hsa-miR-24-3p | 69,287 | 34,032 |
| hsa-miR-99b-5p | 65,508 | 31,322 |
| hsa-miR-101-3p | 64,622 | 45,868 |
| hsa-miR-184 | 58,167 | 151,568 |
| hsa-miR-151a-3p | 53,886 | 57,370 |
| hsa-miR-378a-3p | 53,505 | 11,167 |
| hsa-miR-183-5p | 52,753 | 83,327 |
| hsa-let-7e-5p | 48,850 | 81,004 |
| hsa-miR-103a-3p | 41,454 | 34,228 |
| hsa-let-7i-5p | 40,949 | 66,351 |
| hsa-miR-10a-5p | 37,020 | 14,299 |
| hsa-miR-141-3p | 32,860 | 93,408 |
| hsa-let-7g-5p | 32,464 | 58,038 |
| hsa-miR-181a-5p | 31,491 | 56,942 |
| hsa-miR-30e-5p | 29,462 | 11,512 |
| hsa-miR-92a-3p | 22,367 | 51,442 |
| hsa-miR-129-5p | 22,186 | 10,962 |
| hsa-miR-379-5p | 22,099 | 15,652 |
| hsa-let-7c | 20,411 | 23,638 |
| hsa-miR-191-5p | 20,388 | 61,566 |
| hsa-miR-148b-3p | 17,018 | 22,964 |
| hsa-miR-744-5p | 15,742 | 26,865 |
| hsa-miR-200b-3p | 14,969 | 22,134 |
| hsa-miR-381-3p | 14,546 | 15,696 |
| hsa-miR-26b-5p | 13,910 | 21,614 |
| hsa-miR-29a-3p | 13,379 | 15,815 |
| hsa-miR-23b-3p | 13,276 | 9,987 |
| hsa-miR-200a-3p | 12,885 | 12,698 |
| hsa-miR-27a-3p | 12,470 | 5,378 |
| hsa-miR-411-5p | 12,065 | 16,426 |
| hsa-miR-30a-3p | 11,855 | 3,071 |
| hsa-miR-186-5p | 11,459 | 31,188 |
| hsa-miR-140-5p | 11,017 | 2,453 |
| hsa-miR-10b-5p | 10,614 | 6,933 |
| hsa-miR-16-5p | 10,389 | 18,254 |
| hsa-miR-889 | 10,356 | 15,668 |
| hsa-miR-598 | 9,999 | 3,425 |
| hsa-miR-125b-5p | 9,513 | 16,076 |
| hsa-miR-30e-3p | 9,486 | 6,622 |
| hsa-miR-136-3p | 9,268 | 18,006 |
| hsa-miR-92b-3p | 8,705 | 40,263 |
| hsa-miR-204-5p | 8,346 | 32,817 |
| hsa-miR-410 | 8,043 | 37,884 |
| hsa-miR-409-5p | 7,730 | 29,566 |
| hsa-miR-98-5p | 7,662 | 20,770 |
| hsa-miR-134 | 7,626 | 10,575 |
| hsa-miR-429 | 7,411 | 10,225 |
| hsa-miR-28-5p | 7,262 | 16,560 |
| hsa-miR-194-5p | 7,054 | 10,955 |
| hsa-miR-30c-5p | 6,760 | 3,355 |
| hsa-miR-320a | 6,516 | 11,772 |
| hsa-miR-126-5p | 6,384 | 2,735 |
| hsa-miR-29c-3p | 6,063 | 10,082 |
| hsa-miR-222-3p | 5,874 | 6,233 |
| hsa-miR-221-3p | 5,870 | 10,681 |
| hsa-let-7d-5p | 5,859 | 10,790 |
| hsa-miR-181c-5p | 5,719 | 12,695 |
| hsa-miR-432-5p | 5,680 | 18,508 |
| hsa-miR-126-3p | 5,637 | 888 |
| hsa-miR-132-3p | 5,531 | 7,607 |
| hsa-miR-423-5p | 5,337 | 11,840 |
| hsa-miR-30b-5p | 5,137 | 6,043 |
| hsa-miR-25-3p | 5,015 | 9,954 |
| hsa-miR-23a-3p | 4,993 | 1,533 |
| hsa-miR-199a-5p | 4,342 | 804 |
| hsa-miR-3184-5p | 4,276 | 9,264 |
| hsa-miR-181b-5p | 4,243 | 3,681 |
| hsa-miR-374a-3p | 4,041 | 1,102 |
| hsa-miR-654-5p | 3,994 | 11,054 |
| hsa-miR-217 | 3,723 | 2,066 |
| hsa-miR-382-5p | 3,544 | 6,052 |
| hsa-miR-340-5p | 3,488 | 7,701 |
| hsa-miR-487b | 3,157 | 8,264 |
| hsa-miR-95 | 2,830 | 3,121 |
| hsa-miR-21-3p | 2,576 | 1,222 |
| hsa-miR-370 | 2,379 | 2,188 |
| hsa-miR-128 | 2,352 | 2,847 |
| hsa-miR-136-5p | 2,274 | 3,381 |
| hsa-miR-199b-5p | 2,146 | 395 |
| hsa-miR-9-5p | 2,028 | 687 |
| hsa-miR-93-5p | 2,018 | 914 |
| hsa-miR-15a-5p | 1,982 | 2,914 |
| hsa-miR-301a-3p | 1,918 | 4,742 |
| hsa-miR-100-5p | 1,866 | 447 |
| hsa-miR-153 | 1,755 | 3,025 |
| hsa-miR-376a-5p | 1,710 | 905 |
| hsa-miR-152 | 1,659 | 1,656 |
| hsa-miR-542-5p | 1,601 | 244 |
| hsa-miR-3065-5p | 1,557 | 902 |
| hsa-miR-29b-3p | 1,500 | 1,212 |
| hsa-miR-338-5p | 1,495 | 2,911 |
| hsa-miR-1299 | 1,402 | 321 |
| hsa-miR-486-5p | 1,394 | 4,312 |
| hsa-miR-151b | 1,370 | 3,563 |
| hsa-miR-1468 | 1,336 | 6,172 |
| hsa-miR-19b-3p | 1,321 | 2,081 |
| hsa-miR-216a-5p | 1,275 | 980 |
| hsa-miR-215 | 1,259 | 424 |
| hsa-miR-335-5p | 1,243 | 2,146 |
| hsa-miR-503-5p | 1,107 | 251 |
| hsa-miR-369-5p | 1,101 | 1,340 |
| hsa-miR-210 | 1,056 | 581 |
| hsa-miR-107 | 1,043 | 2,267 |
| hsa-miR-1307-3p | 1,039 | 1,070 |
| hsa-miR-345-5p | 970 | 1,228 |
| hsa-miR-181d | 929 | 954 |
| hsa-miR-874 | 928 | 1,583 |
| hsa-miR-660-5p | 914 | 1,497 |
| hsa-miR-146a-5p | 903 | 825 |
| hsa-miR-139-5p | 865 | 757 |
| hsa-miR-323a-3p | 862 | 1,178 |
| hsa-miR-99a-5p | 854 | 666 |
| hsa-miR-339-5p | 851 | 1,415 |
| hsa-miR-148a-5p | 843 | 4,133 |
| hsa-miR-652-3p | 838 | 954 |
| hsa-miR-145-5p | 804 | 49 |
| hsa-miR-130a-3p | 746 | 401 |
| hsa-miR-376c-3p | 742 | 1,009 |
| hsa-miR-27b-5p | 732 | 1,549 |
| hsa-miR-484 | 723 | 1,050 |
| hsa-miR-424-5p | 722 | 95 |
| hsa-miR-493-5p | 718 | 1,584 |
| hsa-miR-200a-5p | 716 | 760 |
| hsa-miR-421 | 712 | 1,897 |
| hsa-miR-493-3p | 697 | 696 |
| hsa-miR-425-5p | 675 | 757 |
| hsa-miR-135a-5p | 657 | 286 |
| hsa-miR-335-3p | 653 | 751 |
| hsa-miR-1246 | 653 | 0 |
| hsa-miR-106b-5p | 651 | 348 |
| hsa-miR-221-5p | 651 | 579 |
| hsa-miR-320b | 647 | 1,711 |
| hsa-miR-361-5p | 643 | 1,201 |
| hsa-miR-129-1-3p | 639 | 664 |
| hsa-miR-1285-3p | 637 | 386 |
| hsa-miR-200b-5p | 623 | 623 |
| hsa-miR-331-5p | 620 | 357 |
| hsa-miR-99b-3p | 610 | 473 |
| hsa-miR-582-5p | 607 | 801 |
| hsa-miR-433 | 603 | 1,453 |
| hsa-miR-873-5p | 596 | 1,775 |
| hsa-miR-20a-5p | 588 | 77 |
| hsa-miR-342-5p | 586 | 1,017 |
| hsa-miR-203a | 583 | 243 |
| hsa-miR-574-5p | 563 | 829 |
| hsa-miR-125b-2-3p | 553 | 543 |
| hsa-miR-4488 | 530 | 139 |
| hsa-miR-185-5p | 527 | 407 |
| hsa-miR-941 | 524 | 1,207 |
| hsa-miR-212-3p | 520 | 1,382 |
| hsa-miR-195-5p | 516 | 213 |
| hsa-miR-34a-5p | 503 | 190 |
| hsa-miR-96-5p | 499 | 514 |
| hsa-miR-1179 | 499 | 679 |
| hsa-miR-181a-2-3p | 492 | 432 |
| hsa-miR-216b | 490 | 1,086 |
| hsa-miR-141-5p | 487 | 692 |
| hsa-miR-328 | 485 | 974 |
| hsa-miR-4792 | 477 | 79 |
| hsa-miR-30c-2-3p | 473 | 340 |
| hsa-miR-130b-3p | 468 | 2,114 |
| hsa-miR-106b-3p | 459 | 750 |
| hsa-miR-3934-5p | 442 | 99 |
| hsa-miR-17-5p | 437 | 114 |
| hsa-miR-1260b | 436 | 1,326 |
| hsa-miR-1260a | 429 | 1,315 |
| hsa-miR-181c-3p | 393 | 514 |
| hsa-miR-500a-3p | 390 | 977 |
| hsa-miR-1180 | 387 | 592 |
| hsa-miR-323b-5p | 386 | 1,115 |
| hsa-miR-125b-1-3p | 384 | 1,507 |
| hsa-miR-197-3p | 383 | 401 |
| hsa-miR-330-5p | 382 | 607 |
| hsa-let-7d-3p | 382 | 746 |
| hsa-miR-132-5p | 373 | 899 |
| hsa-miR-1 | 369 | 187 |
| hsa-miR-664a-3p | 366 | 527 |
| hsa-miR-374b-5p | 357 | 557 |
| hsa-miR-30d-3p | 356 | 633 |
| hsa-miR-454-3p | 342 | 464 |
| hsa-miR-378c | 337 | 62 |
| hsa-miR-214-3p | 332 | 45 |
| hsa-miR-15b-5p | 330 | 442 |
| hsa-miR-187-3p | 325 | 80 |
| hsa-let-7b-3p | 312 | 151 |
| hsa-miR-181a-3p | 310 | 286 |
| hsa-miR-4532 | 302 | 0 |
| hsa-miR-31-5p | 302 | 0 |
| hsa-miR-155-5p | 301 | 39 |
| hsa-miR-32-5p | 288 | 174 |
| hsa-miR-374a-5p | 281 | 560 |
| hsa-miR-551b-3p | 276 | 381 |
| hsa-miR-29b-2-5p | 272 | 125 |
| hsa-miR-27a-5p | 271 | 177 |
| hsa-miR-340-3p | 261 | 299 |
| hsa-miR-219-5p | 260 | 117 |
| hsa-miR-668 | 258 | 858 |
| hsa-miR-455-5p | 258 | 291 |
| hsa-miR-664a-5p | 251 | 453 |
| hsa-miR-145-3p | 249 | 33 |
| hsa-miR-3960 | 246 | 49 |
| hsa-miR-589-5p | 246 | 371 |
| hsa-miR-3615 | 245 | 517 |
| hsa-miR-4446-5p | 241 | 388 |
| hsa-miR-337-5p | 241 | 123 |
| hsa-miR-4634 | 227 | 0 |
| hsa-miR-19a-3p | 212 | 267 |
| hsa-miR-452-5p | 205 | 0 |
| hsa-miR-494 | 197 | 147 |
| hsa-miR-3200-5p | 192 | 106 |
| hsa-miR-497-5p | 187 | 87 |
| hsa-miR-4286 | 185 | 813 |
| hsa-miR-33b-5p | 181 | 183 |
| hsa-miR-92b-5p | 181 | 248 |
| hsa-miR-376a-3p | 179 | 183 |
| hsa-miR-708-5p | 177 | 245 |
| hsa-miR-1301 | 176 | 286 |
| hsa-miR-4448 | 176 | 160 |
| hsa-miR-1275 | 174 | 780 |
| hsa-miR-1296 | 172 | 345 |
| hsa-miR-450a-5p | 172 | 81 |
| hsa-miR-149-5p | 172 | 308 |
| hsa-miR-324-5p | 171 | 286 |
| hsa-miR-299-5p | 170 | 245 |
| hsa-miR-154-5p | 170 | 214 |
| hsa-miR-885-5p | 170 | 217 |
| hsa-miR-125a-5p | 165 | 204 |
| hsa-miR-137 | 160 | 80 |
| hsa-miR-758-3p | 159 | 383 |
| hsa-let-7a-3p | 157 | 204 |
| hsa-miR-671-5p | 157 | 732 |
| hsa-miR-539-5p | 156 | 163 |
| hsa-miR-532-5p | 153 | 100 |
| hsa-miR-320c | 152 | 266 |
| hsa-miR-377-5p | 151 | 400 |
| hsa-miR-3928 | 147 | 63 |
| hsa-miR-22-5p | 144 | 75 |
| hsa-miR-708-3p | 143 | 629 |
| hsa-miR-431-3p | 137 | 289 |
| hsa-miR-29c-5p | 137 | 79 |
| hsa-miR-363-3p | 135 | 51 |
| hsa-miR-365a-3p | 135 | 121 |
| hsa-miR-495-3p | 134 | 138 |
| hsa-miR-190b | 134 | 432 |
| hsa-let-7f-2-3p | 131 | 280 |
| hsa-miR-329 | 130 | 62 |
| hsa-miR-411-3p | 129 | 97 |
| hsa-miR-502-5p | 129 | 226 |
| hsa-miR-2110 | 128 | 122 |
| hsa-miR-891a | 126 | 0 |
| hsa-miR-577 | 121 | 396 |
| hsa-miR-124-3p | 120 | 41 |
| hsa-miR-485-5p | 116 | 137 |
| hsa-miR-377-3p | 114 | 289 |
| hsa-let-7i-3p | 113 | 58 |
| hsa-miR-489 | 105 | 94 |
| hsa-miR-1292-5p | 104 | 136 |
| hsa-miR-296-5p | 103 | 149 |
| hsa-miR-431-5p | 103 | 211 |
| hsa-miR-146b-5p | 103 | 91 |
| hsa-miR-148b-5p | 100 | 95 |
| hsa-miR-17-3p | 100 | 110 |
| hsa-miR-301b | 99 | 482 |
| hsa-miR-320d | 96 | 166 |
| hsa-miR-138-5p | 93 | 0 |
| hsa-miR-33a-5p | 92 | 0 |
| hsa-let-7e-3p | 92 | 124 |
| hsa-miR-379-3p | 92 | 95 |
| hsa-miR-147b | 91 | 106 |
| hsa-miR-541-3p | 91 | 232 |
| hsa-miR-1197 | 90 | 163 |
| hsa-miR-193b-3p | 86 | 198 |
| hsa-miR-1185-5p | 84 | 142 |
| hsa-miR-133a | 83 | 153 |
| hsa-miR-655 | 81 | 66 |
| hsa-miR-487a | 79 | 179 |
| hsa-miR-501-5p | 79 | 109 |
| hsa-miR-3605-5p | 78 | 211 |
| hsa-miR-543 | 78 | 135 |
| hsa-miR-130b-5p | 74 | 519 |
| hsa-miR-590-5p | 74 | 79 |
| hsa-miR-424-3p | 73 | 40 |
| hsa-miR-4454 | 73 | 64 |
| hsa-miR-4508 | 72 | 0 |
| hsa-miR-30c-1-3p | 71 | 156 |
| hsa-miR-194-3p | 71 | 132 |
| hsa-miR-656 | 71 | 193 |
| hsa-miR-3656 | 68 | 0 |
| hsa-miR-190a | 67 | 0 |
| hsa-miR-3529-5p | 65 | 88 |
| hsa-miR-143-5p | 65 | 0 |
| hsa-miR-7-1-3p | 63 | 82 |
| hsa-miR-1268b | 59 | 0 |
| hsa-miR-1270 | 59 | 108 |
| hsa-miR-224-5p | 58 | 0 |
| hsa-miR-760 | 58 | 225 |
| hsa-miR-378d | 57 | 0 |
| hsa-miR-548k | 55 | 137 |
| hsa-miR-628-5p | 54 | 84 |
| hsa-miR-4492 | 54 | 0 |
| hsa-miR-641 | 54 | 80 |
| hsa-miR-142-5p | 53 | 0 |
| hsa-miR-629-5p | 53 | 55 |
| hsa-miR-218-5p | 52 | 0 |
| hsa-miR-29a-5p | 51 | 52 |
| hsa-miR-3158-5p | 50 | 45 |
| hsa-miR-1287 | 49 | 52 |
| hsa-miR-3909 | 49 | 99 |
| hsa-miR-1268a | 49 | 0 |
| hsa-miR-15b-3p | 46 | 0 |
| hsa-miR-496 | 46 | 77 |
| hsa-miR-380-3p | 46 | 98 |
| hsa-miR-625-5p | 46 | 92 |
| hsa-miR-642a-5p | 46 | 73 |
| hsa-miR-627 | 44 | 0 |
| hsa-miR-99a-3p | 43 | 0 |
| hsa-miR-4662b | 43 | 76 |
| hsa-miR-26b-3p | 43 | 190 |
| hsa-miR-3177-5p | 43 | 91 |
| hsa-miR-451a | 42 | 0 |
| hsa-miR-378f | 42 | 0 |
| hsa-miR-1262 | 41 | 80 |
| hsa-miR-642b-3p | 41 | 62 |
| hsa-miR-1291 | 41 | 0 |
| hsa-miR-205-5p | 40 | 0 |
| hsa-miR-185-3p | 40 | 49 |
| hsa-miR-425-3p | 40 | 63 |
| hsa-miR-3074-5p | 39 | 48 |
| hsa-miR-454-5p | 39 | 86 |
| hsa-miR-150-5p | 39 | 0 |
| hsa-miR-665 | 39 | 129 |
| hsa-miR-1224-5p | 39 | 0 |
| hsa-miR-183-3p | 38 | 105 |
| hsa-miR-135b-5p | 38 | 0 |
| hsa-miR-326 | 37 | 0 |
| hsa-miR-4781-5p | 37 | 0 |
| hsa-miR-105-5p | 37 | 34 |
| hsa-miR-3912 | 36 | 51 |
| hsa-miR-2355-5p | 36 | 0 |
| hsa-miR-3120-5p | 36 | 0 |
| hsa-miR-18a-5p | 36 | 0 |
| hsa-miR-548aa | 35 | 0 |
| hsa-miR-9-3p | 35 | 0 |
| hsa-let-7f-1-3p | 35 | 0 |
| hsa-miR-1276 | 35 | 0 |
| hsa-miR-374b-3p | 35 | 48 |
| hsa-miR-1303 | 34 | 37 |
| hsa-miR-548e | 34 | 38 |
| hsa-miR-4520a-5p | 34 | 58 |
| hsa-miR-23c | 0 | 244 |
| hsa-miR-802 | 0 | 199 |
| hsa-miR-592 | 0 | 133 |
| hsa-miR-541-5p | 0 | 114 |
| hsa-miR-191-3p | 0 | 93 |
| hsa-miR-1193 | 0 | 67 |
| hsa-miR-4677-5p | 0 | 66 |
| hsa-miR-3929 | 0 | 62 |
| hsa-miR-211-5p | 0 | 60 |
| hsa-miR-770-5p | 0 | 49 |
| hsa-miR-744-3p | 0 | 48 |
| hsa-miR-550a-5p | 0 | 46 |
| hsa-miR-92a-1-5p | 0 | 44 |
| hsa-miR-30b-3p | 0 | 42 |
| hsa-miR-7-2-3p | 0 | 42 |
| hsa-miR-192-3p | 0 | 38 |
| hsa-miR-2682-5p | 0 | 38 |
| hsa-miR-93-3p | 0 | 36 |
