# Supplementary material for: The miRNA Profile of Human Pancreatic Islets and Beta-Cells and Relationship to Type 2 Diabetes Pathogenesis
Source: PLoS One. 2013 Jan 25;8(1):e55272. doi: 10.1371/journal.pone.0055272 (PMC3555946; doi:10.1371/journal.pone.0055272)
Supplement: Table S3 — miRNA level comparison between tissues. Each row represents a different miRNA observed for at least 1000 reads across all tissues. The first column has the miRNA identifiers according to miRBase 18. Each other column represents a distinct (set of) tissues used for the comparison. The entries in the columns denote the tissue specificity score as described in the Methods. (DOCX) [file pone.0055272.s003.docx]

| **miRNA** | **Enrichment score islets** | **Enrichment score fat** | **Enrichment score pigment cells** | **Enrichment score liver** | **Enrichment score heart/ muscle** | **Enrichment score orbital gyrus** | **Enrichment score pancreas** | **Enrichment score lung/ spleen/ kidney** | **Enrichment score bone marrow/ CD24+ progenitor cells** | **Enrichment score B-cells/thymus** | **Enrichment score skin** |
| --- | --- | --- | --- | --- | --- | --- | --- | --- | --- | --- | --- |
| hsa-miR-184 | 0.97 | 0.00 | 0.00 | 0.00 | 0.00 | 0.01 | 0.00 | 0.00 | 0.00 | 0.00 | 0.00 |
| hsa-miR-375 | 0.96 | 0.00 | 0.00 | 0.00 | 0.00 | 0.00 | 0.03 | 0.00 | 0.00 | 0.00 | 0.00 |
| hsa-miR-409-5p | 0.93 | 0.01 | 0.00 | 0.01 | 0.00 | 0.02 | 0.00 | 0.01 | 0.01 | 0.00 | 0.02 |
| hsa-miR-182-5p | 0.92 | 0.01 | 0.00 | 0.00 | 0.00 | 0.00 | 0.00 | 0.00 | 0.00 | 0.00 | 0.07 |
| hsa-miR-1468 | 0.91 | 0.01 | 0.01 | 0.01 | 0.00 | 0.01 | 0.02 | 0.01 | 0.01 | 0.01 | 0.00 |
| hsa-miR-183-5p | 0.89 | 0.01 | 0.00 | 0.00 | 0.00 | 0.00 | 0.00 | 0.00 | 0.00 | 0.00 | 0.10 |
| hsa-miR-136-3p | 0.89 | 0.01 | 0.00 | 0.01 | 0.00 | 0.04 | 0.01 | 0.01 | 0.00 | 0.00 | 0.03 |
| hsa-miR-127-5p | 0.86 | 0.00 | 0.00 | 0.01 | 0.00 | 0.07 | 0.01 | 0.01 | 0.01 | 0.00 | 0.03 |
| hsa-miR-370 | 0.83 | 0.03 | 0.00 | 0.00 | 0.00 | 0.07 | 0.00 | 0.00 | 0.01 | 0.00 | 0.04 |
| hsa-miR-153 | 0.83 | 0.04 | 0.00 | 0.00 | 0.00 | 0.09 | 0.01 | 0.00 | 0.00 | 0.00 | 0.01 |
| hsa-miR-200a-5p | 0.82 | 0.00 | 0.00 | 0.00 | 0.00 | 0.00 | 0.01 | 0.00 | 0.01 | 0.01 | 0.14 |
| hsa-miR-7-5p | 0.82 | 0.00 | 0.01 | 0.00 | 0.00 | 0.03 | 0.02 | 0.00 | 0.03 | 0.08 | 0.00 |
| hsa-miR-410 | 0.80 | 0.00 | 0.00 | 0.00 | 0.00 | 0.11 | 0.00 | 0.00 | 0.05 | 0.00 | 0.03 |
| hsa-miR-889 | 0.79 | 0.00 | 0.00 | 0.00 | 0.00 | 0.14 | 0.02 | 0.02 | 0.01 | 0.00 | 0.01 |
| hsa-miR-668 | 0.79 | 0.00 | 0.00 | 0.01 | 0.00 | 0.16 | 0.01 | 0.02 | 0.01 | 0.00 | 0.00 |
| hsa-miR-654-5p | 0.78 | 0.01 | 0.00 | 0.02 | 0.01 | 0.01 | 0.01 | 0.01 | 0.01 | 0.00 | 0.14 |
| hsa-miR-216a | 0.78 | 0.02 | 0.00 | 0.00 | 0.00 | 0.00 | 0.18 | 0.00 | 0.00 | 0.00 | 0.01 |
| hsa-miR-376a-5p | 0.77 | 0.03 | 0.00 | 0.01 | 0.00 | 0.03 | 0.00 | 0.00 | 0.00 | 0.00 | 0.15 |
| hsa-miR-204-5p | 0.77 | 0.01 | 0.00 | 0.00 | 0.00 | 0.10 | 0.01 | 0.10 | 0.00 | 0.00 | 0.01 |
| hsa-miR-381 | 0.76 | 0.02 | 0.00 | 0.00 | 0.00 | 0.00 | 0.00 | 0.00 | 0.00 | 0.00 | 0.22 |
| hsa-miR-411-5p | 0.74 | 0.02 | 0.00 | 0.03 | 0.00 | 0.05 | 0.01 | 0.01 | 0.00 | 0.00 | 0.14 |
| hsa-miR-1179 | 0.73 | 0.13 | 0.00 | 0.00 | 0.01 | 0.05 | 0.04 | 0.01 | 0.01 | 0.01 | 0.01 |
| hsa-miR-493-5p | 0.71 | 0.01 | 0.01 | 0.02 | 0.00 | 0.00 | 0.01 | 0.02 | 0.05 | 0.00 | 0.17 |
| hsa-miR-212-3p | 0.69 | 0.02 | 0.04 | 0.01 | 0.00 | 0.11 | 0.00 | 0.04 | 0.02 | 0.03 | 0.02 |
| hsa-miR-132-3p | 0.66 | 0.06 | 0.02 | 0.01 | 0.01 | 0.07 | 0.01 | 0.03 | 0.01 | 0.04 | 0.07 |
| hsa-miR-141-3p | 0.66 | 0.00 | 0.00 | 0.00 | 0.00 | 0.00 | 0.01 | 0.00 | 0.00 | 0.00 | 0.33 |
| hsa-miR-493-3p | 0.63 | 0.02 | 0.01 | 0.03 | 0.01 | 0.01 | 0.02 | 0.05 | 0.02 | 0.00 | 0.20 |
| hsa-miR-1180 | 0.62 | 0.04 | 0.00 | 0.01 | 0.01 | 0.17 | 0.01 | 0.07 | 0.01 | 0.01 | 0.06 |
| hsa-miR-429 | 0.60 | 0.00 | 0.00 | 0.01 | 0.00 | 0.00 | 0.05 | 0.02 | 0.00 | 0.00 | 0.32 |
| hsa-miR-27b-3p | 0.60 | 0.05 | 0.01 | 0.00 | 0.01 | 0.00 | 0.00 | 0.01 | 0.01 | 0.00 | 0.31 |
| hsa-miR-148a-5p | 0.60 | 0.06 | 0.01 | 0.02 | 0.00 | 0.00 | 0.02 | 0.00 | 0.00 | 0.02 | 0.27 |
| hsa-miR-125b-1-3p | 0.60 | 0.05 | 0.04 | 0.02 | 0.01 | 0.15 | 0.02 | 0.02 | 0.00 | 0.01 | 0.09 |
| hsa-miR-582-5p | 0.59 | 0.02 | 0.24 | 0.01 | 0.00 | 0.01 | 0.00 | 0.00 | 0.04 | 0.01 | 0.06 |
| hsa-miR-129-5p | 0.58 | 0.00 | 0.06 | 0.00 | 0.00 | 0.33 | 0.02 | 0.00 | 0.00 | 0.00 | 0.00 |
| hsa-miR-671-5p | 0.56 | 0.04 | 0.11 | 0.02 | 0.00 | 0.00 | 0.00 | 0.01 | 0.12 | 0.10 | 0.04 |
| hsa-miR-200a-3p | 0.55 | 0.00 | 0.00 | 0.03 | 0.00 | 0.00 | 0.10 | 0.03 | 0.00 | 0.01 | 0.29 |
| hsa-miR-148a-3p | 0.53 | 0.04 | 0.01 | 0.07 | 0.00 | 0.00 | 0.06 | 0.01 | 0.00 | 0.04 | 0.24 |
| hsa-miR-181c-5p | 0.52 | 0.04 | 0.02 | 0.01 | 0.01 | 0.13 | 0.01 | 0.01 | 0.13 | 0.05 | 0.08 |
| hsa-miR-323b-5p | 0.52 | 0.00 | 0.01 | 0.01 | 0.01 | 0.33 | 0.06 | 0.01 | 0.05 | 0.00 | 0.00 |
| hsa-miR-141-5p | 0.51 | 0.00 | 0.00 | 0.00 | 0.00 | 0.00 | 0.02 | 0.00 | 0.00 | 0.00 | 0.46 |
| hsa-miR-369-5p | 0.47 | 0.04 | 0.01 | 0.07 | 0.01 | 0.18 | 0.05 | 0.03 | 0.00 | 0.00 | 0.14 |
| hsa-miR-190b | 0.46 | 0.07 | 0.00 | 0.04 | 0.03 | 0.00 | 0.24 | 0.07 | 0.02 | 0.04 | 0.03 |
| hsa-miR-134 | 0.45 | 0.01 | 0.00 | 0.01 | 0.01 | 0.31 | 0.04 | 0.08 | 0.04 | 0.00 | 0.04 |
| hsa-miR-194-5p | 0.44 | 0.03 | 0.00 | 0.38 | 0.00 | 0.00 | 0.02 | 0.03 | 0.01 | 0.02 | 0.06 |
| hsa-miR-3065-5p | 0.44 | 0.05 | 0.01 | 0.01 | 0.00 | 0.30 | 0.02 | 0.02 | 0.01 | 0.00 | 0.13 |
| hsa-miR-340-3p | 0.44 | 0.45 | 0.01 | 0.01 | 0.00 | 0.00 | 0.00 | 0.01 | 0.01 | 0.01 | 0.08 |
| hsa-miR-132-5p | 0.44 | 0.07 | 0.03 | 0.02 | 0.01 | 0.31 | 0.03 | 0.03 | 0.01 | 0.05 | 0.02 |
| hsa-miR-148b-3p | 0.43 | 0.11 | 0.06 | 0.03 | 0.00 | 0.02 | 0.01 | 0.02 | 0.04 | 0.07 | 0.23 |
| hsa-miR-744-5p | 0.42 | 0.03 | 0.10 | 0.01 | 0.01 | 0.13 | 0.01 | 0.02 | 0.10 | 0.12 | 0.04 |
| hsa-miR-720 | 0.42 | 0.08 | 0.01 | 0.02 | 0.00 | 0.00 | 0.00 | 0.00 | 0.01 | 0.09 | 0.37 |
| hsa-miR-200b-3p | 0.40 | 0.00 | 0.00 | 0.03 | 0.00 | 0.00 | 0.08 | 0.02 | 0.00 | 0.00 | 0.47 |
| hsa-miR-577 | 0.39 | 0.01 | 0.02 | 0.00 | 0.00 | 0.27 | 0.11 | 0.03 | 0.01 | 0.08 | 0.07 |
| hsa-mir-151 | 0.38 | 0.06 | 0.15 | 0.08 | 0.02 | 0.05 | 0.04 | 0.03 | 0.02 | 0.06 | 0.10 |
| hsa-miR-301a-3p | 0.37 | 0.19 | 0.05 | 0.02 | 0.01 | 0.01 | 0.01 | 0.01 | 0.25 | 0.08 | 0.03 |
| hsa-miR-95 | 0.37 | 0.12 | 0.00 | 0.03 | 0.08 | 0.08 | 0.03 | 0.04 | 0.00 | 0.05 | 0.21 |
| hsa-miR-30d-3p | 0.37 | 0.18 | 0.08 | 0.03 | 0.04 | 0.01 | 0.03 | 0.02 | 0.02 | 0.07 | 0.14 |
| hsa-miR-1260a | 0.36 | 0.08 | 0.01 | 0.01 | 0.01 | 0.01 | 0.01 | 0.02 | 0.01 | 0.02 | 0.46 |
| hsa-miR-1260b | 0.35 | 0.06 | 0.01 | 0.01 | 0.01 | 0.01 | 0.02 | 0.01 | 0.01 | 0.05 | 0.45 |
| hsa-miR-130b-5p | 0.35 | 0.03 | 0.14 | 0.04 | 0.01 | 0.01 | 0.03 | 0.00 | 0.10 | 0.24 | 0.06 |
| hsa-miR-1299 | 0.33 | 0.03 | 0.03 | 0.01 | 0.11 | 0.25 | 0.07 | 0.12 | 0.01 | 0.03 | 0.01 |
| hsa-miR-432-5p | 0.33 | 0.00 | 0.00 | 0.02 | 0.01 | 0.52 | 0.04 | 0.06 | 0.01 | 0.00 | 0.00 |
| hsa-miR-4446-5p | 0.33 | 0.01 | 0.00 | 0.01 | 0.01 | 0.16 | 0.00 | 0.02 | 0.21 | 0.26 | 0.01 |
| hsa-miR-92b-3p | 0.32 | 0.00 | 0.13 | 0.01 | 0.01 | 0.30 | 0.03 | 0.10 | 0.06 | 0.02 | 0.02 |
| hsa-miR-98 | 0.32 | 0.08 | 0.05 | 0.02 | 0.05 | 0.10 | 0.05 | 0.07 | 0.06 | 0.12 | 0.08 |
| hsa-miR-99b-5p | 0.32 | 0.09 | 0.17 | 0.02 | 0.02 | 0.10 | 0.03 | 0.05 | 0.01 | 0.00 | 0.18 |
| hsa-miR-4286 | 0.31 | 0.11 | 0.01 | 0.06 | 0.01 | 0.02 | 0.03 | 0.03 | 0.04 | 0.04 | 0.33 |
| hsa-miR-484 | 0.31 | 0.11 | 0.06 | 0.05 | 0.02 | 0.02 | 0.01 | 0.06 | 0.08 | 0.13 | 0.15 |
| hsa-miR-377-5p | 0.30 | 0.02 | 0.02 | 0.24 | 0.00 | 0.25 | 0.05 | 0.04 | 0.03 | 0.00 | 0.05 |
| hsa-miR-135a-5p | 0.30 | 0.01 | 0.00 | 0.01 | 0.01 | 0.39 | 0.14 | 0.10 | 0.01 | 0.01 | 0.01 |
| hsa-miR-338-5p | 0.30 | 0.14 | 0.00 | 0.02 | 0.01 | 0.24 | 0.02 | 0.07 | 0.06 | 0.00 | 0.13 |
| hsa-miR-30d-5p | 0.28 | 0.06 | 0.07 | 0.11 | 0.08 | 0.03 | 0.03 | 0.07 | 0.02 | 0.06 | 0.19 |
| hsa-miR-500a-3p | 0.28 | 0.06 | 0.23 | 0.04 | 0.02 | 0.02 | 0.02 | 0.04 | 0.05 | 0.07 | 0.16 |
| hsa-miR-487b | 0.28 | 0.01 | 0.00 | 0.03 | 0.00 | 0.54 | 0.03 | 0.05 | 0.05 | 0.00 | 0.01 |
| hsa-miR-1275 | 0.28 | 0.03 | 0.01 | 0.03 | 0.01 | 0.06 | 0.11 | 0.05 | 0.06 | 0.26 | 0.10 |
| hsa-miR-382-5p | 0.28 | 0.01 | 0.00 | 0.02 | 0.01 | 0.51 | 0.05 | 0.05 | 0.04 | 0.00 | 0.03 |
| hsa-miR-136-5p | 0.27 | 0.23 | 0.00 | 0.02 | 0.02 | 0.27 | 0.06 | 0.06 | 0.01 | 0.00 | 0.06 |
| hsa-miR-200b-5p | 0.26 | 0.00 | 0.00 | 0.07 | 0.00 | 0.00 | 0.17 | 0.03 | 0.00 | 0.01 | 0.45 |
| hsa-miR-708-3p | 0.25 | 0.23 | 0.01 | 0.02 | 0.01 | 0.23 | 0.01 | 0.01 | 0.01 | 0.01 | 0.21 |
| hsa-miR-589-5p | 0.25 | 0.05 | 0.15 | 0.01 | 0.01 | 0.08 | 0.04 | 0.05 | 0.08 | 0.23 | 0.05 |
| hsa-miR-379-5p | 0.24 | 0.09 | 0.00 | 0.03 | 0.01 | 0.15 | 0.01 | 0.02 | 0.01 | 0.00 | 0.44 |
| hsa-miR-96-5p | 0.24 | 0.08 | 0.00 | 0.00 | 0.00 | 0.00 | 0.05 | 0.03 | 0.01 | 0.02 | 0.56 |
| hsa-miR-421 | 0.24 | 0.04 | 0.11 | 0.03 | 0.03 | 0.09 | 0.01 | 0.03 | 0.26 | 0.13 | 0.03 |
| hsa-miR-361-5p | 0.24 | 0.05 | 0.07 | 0.03 | 0.01 | 0.03 | 0.01 | 0.04 | 0.08 | 0.35 | 0.08 |
| hsa-miR-423-5p | 0.24 | 0.10 | 0.19 | 0.03 | 0.01 | 0.02 | 0.01 | 0.02 | 0.12 | 0.16 | 0.11 |
| hsa-miR-26a-5p | 0.23 | 0.08 | 0.03 | 0.05 | 0.03 | 0.04 | 0.03 | 0.07 | 0.10 | 0.13 | 0.22 |
| hsa-miR-186-5p | 0.23 | 0.16 | 0.03 | 0.07 | 0.02 | 0.03 | 0.04 | 0.05 | 0.05 | 0.13 | 0.19 |
| hsa-miR-3184-5p | 0.22 | 0.09 | 0.21 | 0.03 | 0.01 | 0.02 | 0.01 | 0.02 | 0.14 | 0.15 | 0.10 |
| hsa-miR-542-5p | 0.22 | 0.25 | 0.10 | 0.03 | 0.04 | 0.01 | 0.01 | 0.05 | 0.11 | 0.04 | 0.15 |
| hsa-miR-28-5p | 0.22 | 0.05 | 0.10 | 0.17 | 0.05 | 0.02 | 0.03 | 0.04 | 0.04 | 0.17 | 0.11 |
| hsa-miR-27b-5p | 0.22 | 0.10 | 0.04 | 0.17 | 0.16 | 0.03 | 0.03 | 0.03 | 0.01 | 0.01 | 0.20 |
| hsa-mir-323 | 0.21 | 0.00 | 0.00 | 0.02 | 0.01 | 0.70 | 0.02 | 0.02 | 0.01 | 0.00 | 0.01 |
| hsa-miR-22-3p | 0.21 | 0.26 | 0.07 | 0.11 | 0.11 | 0.04 | 0.04 | 0.07 | 0.01 | 0.01 | 0.07 |
| hsa-miR-873-5p | 0.20 | 0.00 | 0.07 | 0.00 | 0.00 | 0.68 | 0.02 | 0.02 | 0.01 | 0.00 | 0.01 |
| hsa-let-7d-3p | 0.19 | 0.05 | 0.08 | 0.07 | 0.06 | 0.18 | 0.06 | 0.10 | 0.02 | 0.02 | 0.17 |
| hsa-miR-154-5p | 0.18 | 0.01 | 0.00 | 0.06 | 0.04 | 0.42 | 0.08 | 0.13 | 0.01 | 0.00 | 0.07 |
| hsa-miR-874 | 0.18 | 0.02 | 0.00 | 0.01 | 0.02 | 0.65 | 0.01 | 0.05 | 0.01 | 0.02 | 0.03 |
| hsa-miR-551b-3p | 0.18 | 0.06 | 0.00 | 0.03 | 0.03 | 0.19 | 0.14 | 0.05 | 0.29 | 0.01 | 0.02 |
| hsa-miR-151b | 0.17 | 0.11 | 0.23 | 0.09 | 0.02 | 0.07 | 0.02 | 0.04 | 0.03 | 0.13 | 0.09 |
| hsa-miR-30b-5p | 0.17 | 0.38 | 0.01 | 0.02 | 0.01 | 0.01 | 0.01 | 0.02 | 0.01 | 0.02 | 0.34 |
| hsa-miR-339-5p | 0.17 | 0.14 | 0.07 | 0.03 | 0.04 | 0.11 | 0.08 | 0.09 | 0.10 | 0.08 | 0.08 |
| hsa-miR-376c | 0.17 | 0.06 | 0.00 | 0.15 | 0.01 | 0.21 | 0.05 | 0.10 | 0.07 | 0.00 | 0.18 |
| hsa-miR-200c-3p | 0.17 | 0.00 | 0.00 | 0.00 | 0.00 | 0.00 | 0.07 | 0.01 | 0.00 | 0.00 | 0.75 |
| hsa-miR-335-3p | 0.16 | 0.68 | 0.00 | 0.01 | 0.00 | 0.01 | 0.01 | 0.01 | 0.00 | 0.01 | 0.11 |
| hsa-miR-3615 | 0.15 | 0.04 | 0.02 | 0.01 | 0.01 | 0.01 | 0.01 | 0.02 | 0.40 | 0.29 | 0.03 |
| hsa-miR-125b-2-3p | 0.15 | 0.19 | 0.00 | 0.02 | 0.01 | 0.03 | 0.00 | 0.00 | 0.00 | 0.00 | 0.58 |
| hsa-miR-494 | 0.15 | 0.12 | 0.01 | 0.11 | 0.02 | 0.13 | 0.03 | 0.11 | 0.09 | 0.00 | 0.23 |
| hsa-miR-197-3p | 0.15 | 0.11 | 0.06 | 0.01 | 0.02 | 0.04 | 0.01 | 0.04 | 0.08 | 0.10 | 0.37 |
| hsa-miR-29b-2-5p | 0.14 | 0.33 | 0.00 | 0.03 | 0.02 | 0.17 | 0.03 | 0.06 | 0.06 | 0.05 | 0.11 |
| hsa-miR-454-3p | 0.14 | 0.30 | 0.05 | 0.01 | 0.01 | 0.00 | 0.01 | 0.02 | 0.08 | 0.15 | 0.24 |
| hsa-miR-598 | 0.14 | 0.01 | 0.01 | 0.01 | 0.01 | 0.66 | 0.03 | 0.07 | 0.03 | 0.01 | 0.03 |
| hsa-miR-328 | 0.14 | 0.03 | 0.02 | 0.02 | 0.05 | 0.54 | 0.04 | 0.07 | 0.01 | 0.01 | 0.07 |
| hsa-miR-664-3p | 0.13 | 0.02 | 0.04 | 0.04 | 0.04 | 0.07 | 0.04 | 0.08 | 0.13 | 0.19 | 0.21 |
| hsa-miR-106b-3p | 0.13 | 0.03 | 0.16 | 0.10 | 0.02 | 0.03 | 0.01 | 0.03 | 0.20 | 0.22 | 0.07 |
| hsa-miR-574-5p | 0.13 | 0.05 | 0.08 | 0.15 | 0.02 | 0.03 | 0.06 | 0.09 | 0.01 | 0.00 | 0.38 |
| hsa-miR-30e-3p | 0.13 | 0.08 | 0.22 | 0.04 | 0.02 | 0.02 | 0.01 | 0.02 | 0.07 | 0.23 | 0.17 |
| hsa-miR-181c-3p | 0.13 | 0.02 | 0.02 | 0.00 | 0.01 | 0.40 | 0.04 | 0.02 | 0.21 | 0.10 | 0.05 |
| hsa-miR-99b-3p | 0.12 | 0.02 | 0.18 | 0.03 | 0.07 | 0.30 | 0.06 | 0.14 | 0.05 | 0.00 | 0.02 |
| hsa-miR-21-3p | 0.12 | 0.00 | 0.39 | 0.25 | 0.00 | 0.00 | 0.01 | 0.03 | 0.08 | 0.08 | 0.01 |
| hsa-miR-374a-3p | 0.12 | 0.23 | 0.04 | 0.15 | 0.04 | 0.07 | 0.05 | 0.06 | 0.01 | 0.10 | 0.13 |
| hsa-miR-143-3p | 0.11 | 0.54 | 0.00 | 0.01 | 0.01 | 0.00 | 0.01 | 0.01 | 0.00 | 0.00 | 0.31 |
| hsa-miR-340-5p | 0.11 | 0.03 | 0.27 | 0.02 | 0.03 | 0.17 | 0.03 | 0.05 | 0.21 | 0.06 | 0.01 |
| hsa-miR-30e-5p | 0.11 | 0.19 | 0.02 | 0.06 | 0.04 | 0.02 | 0.02 | 0.03 | 0.04 | 0.08 | 0.40 |
| hsa-miR-149-5p | 0.11 | 0.06 | 0.00 | 0.00 | 0.00 | 0.03 | 0.00 | 0.00 | 0.00 | 0.00 | 0.79 |
| hsa-miR-30a-5p | 0.11 | 0.16 | 0.07 | 0.10 | 0.08 | 0.04 | 0.06 | 0.18 | 0.00 | 0.00 | 0.20 |
| hsa-miR-27a-5p | 0.11 | 0.04 | 0.08 | 0.01 | 0.00 | 0.00 | 0.00 | 0.00 | 0.15 | 0.02 | 0.59 |
| hsa-miR-652-3p | 0.11 | 0.45 | 0.02 | 0.01 | 0.04 | 0.04 | 0.02 | 0.04 | 0.05 | 0.04 | 0.16 |
| hsa-miR-324-5p | 0.10 | 0.17 | 0.04 | 0.05 | 0.03 | 0.22 | 0.02 | 0.11 | 0.08 | 0.07 | 0.10 |
| hsa-miR-4488 | 0.10 | 0.05 | 0.15 | 0.11 | 0.07 | 0.02 | 0.11 | 0.20 | 0.04 | 0.04 | 0.09 |
| hsa-miR-1285-3p | 0.10 | 0.05 | 0.19 | 0.11 | 0.02 | 0.01 | 0.03 | 0.03 | 0.15 | 0.17 | 0.14 |
| hsa-miR-23b-3p | 0.10 | 0.04 | 0.03 | 0.18 | 0.08 | 0.04 | 0.03 | 0.07 | 0.02 | 0.01 | 0.40 |
| hsa-miR-3934 | 0.10 | 0.02 | 0.10 | 0.01 | 0.02 | 0.00 | 0.02 | 0.06 | 0.09 | 0.51 | 0.07 |
| hsa-miR-532-5p | 0.10 | 0.05 | 0.09 | 0.10 | 0.03 | 0.04 | 0.03 | 0.13 | 0.09 | 0.07 | 0.26 |
| hsa-miR-3960 | 0.10 | 0.03 | 0.02 | 0.03 | 0.12 | 0.03 | 0.10 | 0.47 | 0.03 | 0.01 | 0.07 |
| hsa-miR-33b-5p | 0.10 | 0.25 | 0.04 | 0.07 | 0.01 | 0.26 | 0.05 | 0.03 | 0.12 | 0.04 | 0.03 |
| hsa-miR-187-3p | 0.09 | 0.00 | 0.00 | 0.04 | 0.02 | 0.24 | 0.06 | 0.39 | 0.05 | 0.01 | 0.11 |
| hsa-let-7a-3p | 0.09 | 0.07 | 0.03 | 0.08 | 0.04 | 0.12 | 0.08 | 0.07 | 0.03 | 0.09 | 0.29 |
| hsa-miR-125b-5p | 0.09 | 0.18 | 0.01 | 0.07 | 0.03 | 0.15 | 0.02 | 0.03 | 0.00 | 0.00 | 0.43 |
| hsa-let-7b-3p | 0.09 | 0.06 | 0.04 | 0.07 | 0.01 | 0.08 | 0.07 | 0.07 | 0.03 | 0.03 | 0.46 |
| hsa-miR-30a-3p | 0.09 | 0.19 | 0.26 | 0.04 | 0.05 | 0.05 | 0.05 | 0.15 | 0.00 | 0.00 | 0.13 |
| hsa-miR-30c-1-3p | 0.08 | 0.02 | 0.12 | 0.16 | 0.08 | 0.10 | 0.05 | 0.03 | 0.05 | 0.25 | 0.07 |
| hsa-miR-146b-5p | 0.08 | 0.02 | 0.00 | 0.31 | 0.01 | 0.05 | 0.07 | 0.12 | 0.21 | 0.10 | 0.04 |
| hsa-miR-192-5p | 0.08 | 0.00 | 0.00 | 0.81 | 0.00 | 0.00 | 0.06 | 0.02 | 0.01 | 0.02 | 0.00 |
| hsa-miR-129-1-3p | 0.08 | 0.00 | 0.02 | 0.00 | 0.00 | 0.85 | 0.04 | 0.00 | 0.00 | 0.00 | 0.00 |
| hsa-miR-590-5p | 0.08 | 0.45 | 0.05 | 0.05 | 0.03 | 0.02 | 0.02 | 0.01 | 0.02 | 0.12 | 0.14 |
| hsa-miR-941 | 0.08 | 0.01 | 0.15 | 0.04 | 0.03 | 0.04 | 0.02 | 0.04 | 0.31 | 0.24 | 0.02 |
| hsa-miR-628-5p | 0.08 | 0.13 | 0.01 | 0.04 | 0.14 | 0.41 | 0.04 | 0.08 | 0.03 | 0.02 | 0.03 |
| hsa-let-7e-5p | 0.08 | 0.02 | 0.47 | 0.05 | 0.06 | 0.14 | 0.06 | 0.08 | 0.02 | 0.01 | 0.01 |
| hsa-miR-450a-5p | 0.08 | 0.28 | 0.02 | 0.08 | 0.08 | 0.01 | 0.01 | 0.06 | 0.15 | 0.09 | 0.14 |
| hsa-miR-23c | 0.08 | 0.02 | 0.10 | 0.34 | 0.04 | 0.07 | 0.14 | 0.11 | 0.07 | 0.02 | 0.03 |
| hsa-miR-1185-5p | 0.08 | 0.06 | 0.01 | 0.10 | 0.01 | 0.62 | 0.04 | 0.03 | 0.02 | 0.00 | 0.04 |
| hsa-miR-376a-3p | 0.08 | 0.06 | 0.01 | 0.07 | 0.02 | 0.19 | 0.07 | 0.14 | 0.04 | 0.00 | 0.33 |
| hsa-miR-660-5p | 0.07 | 0.07 | 0.12 | 0.07 | 0.03 | 0.03 | 0.04 | 0.22 | 0.08 | 0.13 | 0.15 |
| hsa-miR-21-5p | 0.07 | 0.08 | 0.09 | 0.09 | 0.01 | 0.01 | 0.06 | 0.16 | 0.12 | 0.13 | 0.18 |
| hsa-miR-625-5p | 0.07 | 0.05 | 0.20 | 0.05 | 0.02 | 0.04 | 0.04 | 0.02 | 0.08 | 0.37 | 0.06 |
| hsa-miR-92a-3p | 0.07 | 0.02 | 0.29 | 0.02 | 0.01 | 0.01 | 0.01 | 0.03 | 0.15 | 0.32 | 0.09 |
| hsa-miR-433 | 0.07 | 0.00 | 0.00 | 0.02 | 0.01 | 0.85 | 0.02 | 0.01 | 0.01 | 0.00 | 0.00 |
| hsa-miR-1301 | 0.07 | 0.01 | 0.27 | 0.01 | 0.04 | 0.28 | 0.01 | 0.09 | 0.14 | 0.05 | 0.01 |
| hsa-miR-32-5p | 0.07 | 0.37 | 0.10 | 0.03 | 0.01 | 0.01 | 0.01 | 0.03 | 0.10 | 0.05 | 0.22 |
| hsa-miR-1270 | 0.07 | 0.04 | 0.02 | 0.01 | 0.04 | 0.04 | 0.04 | 0.04 | 0.28 | 0.36 | 0.07 |
| hsa-miR-10a-5p | 0.07 | 0.12 | 0.09 | 0.04 | 0.02 | 0.00 | 0.07 | 0.25 | 0.04 | 0.00 | 0.32 |
| hsa-miR-30c-2-3p | 0.07 | 0.16 | 0.15 | 0.17 | 0.05 | 0.09 | 0.10 | 0.14 | 0.00 | 0.00 | 0.08 |
| hsa-miR-30c-5p | 0.06 | 0.36 | 0.01 | 0.02 | 0.01 | 0.01 | 0.00 | 0.02 | 0.02 | 0.04 | 0.44 |
| hsa-miR-191-5p | 0.06 | 0.03 | 0.12 | 0.11 | 0.01 | 0.01 | 0.01 | 0.02 | 0.29 | 0.27 | 0.06 |
| hsa-miR-210 | 0.06 | 0.05 | 0.06 | 0.13 | 0.03 | 0.03 | 0.01 | 0.06 | 0.05 | 0.15 | 0.37 |
| hsa-miR-641 | 0.06 | 0.01 | 0.10 | 0.18 | 0.01 | 0.03 | 0.06 | 0.02 | 0.13 | 0.41 | 0.00 |
| hsa-miR-101-3p | 0.06 | 0.06 | 0.05 | 0.34 | 0.06 | 0.09 | 0.04 | 0.07 | 0.06 | 0.06 | 0.11 |
| hsa-miR-345-5p | 0.06 | 0.05 | 0.20 | 0.14 | 0.05 | 0.07 | 0.02 | 0.06 | 0.26 | 0.05 | 0.04 |
| hsa-miR-7-1-3p | 0.06 | 0.02 | 0.04 | 0.06 | 0.02 | 0.17 | 0.04 | 0.03 | 0.09 | 0.38 | 0.09 |
| hsa-let-7i-3p | 0.06 | 0.10 | 0.19 | 0.02 | 0.03 | 0.06 | 0.02 | 0.08 | 0.06 | 0.06 | 0.31 |
| hsa-miR-455-5p | 0.06 | 0.03 | 0.01 | 0.57 | 0.01 | 0.01 | 0.02 | 0.07 | 0.00 | 0.01 | 0.22 |
| hsa-miR-34a-5p | 0.06 | 0.17 | 0.03 | 0.02 | 0.02 | 0.08 | 0.04 | 0.21 | 0.01 | 0.00 | 0.35 |
| hsa-miR-16-5p | 0.06 | 0.06 | 0.04 | 0.03 | 0.01 | 0.01 | 0.01 | 0.04 | 0.39 | 0.26 | 0.12 |
| hsa-miR-1307-3p | 0.06 | 0.03 | 0.13 | 0.08 | 0.06 | 0.20 | 0.03 | 0.15 | 0.10 | 0.13 | 0.03 |
| hsa-miR-329 | 0.06 | 0.01 | 0.01 | 0.03 | 0.00 | 0.83 | 0.01 | 0.02 | 0.01 | 0.00 | 0.04 |
| hsa-miR-502-5p | 0.05 | 0.09 | 0.29 | 0.08 | 0.04 | 0.03 | 0.04 | 0.09 | 0.11 | 0.08 | 0.10 |
| hsa-miR-374a-5p | 0.05 | 0.25 | 0.04 | 0.17 | 0.03 | 0.05 | 0.05 | 0.08 | 0.04 | 0.15 | 0.09 |
| hsa-miR-29a-5p | 0.05 | 0.20 | 0.03 | 0.09 | 0.07 | 0.21 | 0.07 | 0.12 | 0.02 | 0.02 | 0.12 |
| hsa-miR-19b-3p | 0.05 | 0.38 | 0.02 | 0.03 | 0.02 | 0.02 | 0.05 | 0.09 | 0.13 | 0.11 | 0.10 |
| hsa-miR-181a-3p | 0.05 | 0.02 | 0.04 | 0.01 | 0.01 | 0.15 | 0.01 | 0.03 | 0.08 | 0.57 | 0.03 |
| hsa-miR-629-5p | 0.05 | 0.30 | 0.06 | 0.01 | 0.00 | 0.00 | 0.01 | 0.02 | 0.11 | 0.28 | 0.15 |
| hsa-miR-181a-5p | 0.05 | 0.03 | 0.10 | 0.01 | 0.01 | 0.15 | 0.01 | 0.03 | 0.10 | 0.45 | 0.05 |
| hsa-miR-181a-2-3p | 0.05 | 0.02 | 0.36 | 0.03 | 0.06 | 0.07 | 0.03 | 0.03 | 0.04 | 0.17 | 0.15 |
| hsa-miR-543 | 0.05 | 0.00 | 0.02 | 0.08 | 0.02 | 0.59 | 0.05 | 0.06 | 0.10 | 0.00 | 0.02 |
| hsa-miR-26b-5p | 0.04 | 0.05 | 0.02 | 0.10 | 0.04 | 0.03 | 0.06 | 0.15 | 0.17 | 0.23 | 0.11 |
| hsa-miR-15a-5p | 0.04 | 0.11 | 0.02 | 0.02 | 0.01 | 0.02 | 0.01 | 0.05 | 0.32 | 0.29 | 0.12 |
| hsa-miR-3150b-5p | 0.04 | 0.02 | 0.06 | 0.04 | 0.00 | 0.00 | 0.00 | 0.05 | 0.38 | 0.39 | 0.02 |
| hsa-miR-4508 | 0.04 | 0.03 | 0.02 | 0.10 | 0.05 | 0.02 | 0.18 | 0.30 | 0.08 | 0.03 | 0.16 |
| hsa-let-7a-5p | 0.04 | 0.07 | 0.18 | 0.06 | 0.07 | 0.09 | 0.11 | 0.09 | 0.05 | 0.13 | 0.11 |
| hsa-miR-29c-3p | 0.04 | 0.05 | 0.01 | 0.16 | 0.10 | 0.15 | 0.10 | 0.14 | 0.10 | 0.12 | 0.03 |
| hsa-miR-486-5p | 0.04 | 0.13 | 0.00 | 0.02 | 0.27 | 0.02 | 0.06 | 0.29 | 0.05 | 0.01 | 0.10 |
| hsa-miR-425-5p | 0.04 | 0.26 | 0.02 | 0.03 | 0.01 | 0.04 | 0.01 | 0.03 | 0.18 | 0.12 | 0.26 |
| hsa-miR-27a-3p | 0.04 | 0.16 | 0.02 | 0.01 | 0.01 | 0.00 | 0.00 | 0.01 | 0.02 | 0.01 | 0.72 |
| hsa-let-7f-5p | 0.04 | 0.02 | 0.20 | 0.03 | 0.08 | 0.08 | 0.07 | 0.10 | 0.12 | 0.22 | 0.03 |
| hsa-miR-103a-3p | 0.04 | 0.02 | 0.23 | 0.10 | 0.02 | 0.11 | 0.02 | 0.05 | 0.13 | 0.23 | 0.05 |
| hsa-miR-374b-5p | 0.04 | 0.16 | 0.10 | 0.10 | 0.04 | 0.07 | 0.04 | 0.09 | 0.09 | 0.16 | 0.12 |
| hsa-miR-10b-5p | 0.04 | 0.23 | 0.04 | 0.00 | 0.00 | 0.00 | 0.00 | 0.02 | 0.00 | 0.00 | 0.67 |
| hsa-miR-2110 | 0.04 | 0.02 | 0.05 | 0.07 | 0.10 | 0.09 | 0.05 | 0.14 | 0.23 | 0.18 | 0.03 |
| hsa-miR-24-3p | 0.04 | 0.20 | 0.03 | 0.01 | 0.03 | 0.01 | 0.01 | 0.04 | 0.04 | 0.01 | 0.58 |
| hsa-miR-222-3p | 0.04 | 0.02 | 0.29 | 0.05 | 0.03 | 0.13 | 0.01 | 0.05 | 0.23 | 0.10 | 0.05 |
| hsa-miR-19a-3p | 0.03 | 0.80 | 0.01 | 0.01 | 0.00 | 0.00 | 0.01 | 0.01 | 0.02 | 0.04 | 0.07 |
| hsa-miR-185-3p | 0.03 | 0.02 | 0.53 | 0.06 | 0.05 | 0.12 | 0.01 | 0.03 | 0.07 | 0.06 | 0.02 |
| hsa-miR-217 | 0.03 | 0.00 | 0.00 | 0.00 | 0.00 | 0.00 | 0.96 | 0.00 | 0.00 | 0.00 | 0.00 |
| hsa-miR-548k | 0.03 | 0.02 | 0.24 | 0.09 | 0.04 | 0.02 | 0.01 | 0.03 | 0.20 | 0.28 | 0.01 |
| hsa-miR-331-5p | 0.03 | 0.01 | 0.07 | 0.04 | 0.09 | 0.35 | 0.02 | 0.14 | 0.03 | 0.19 | 0.03 |
| hsa-let-7i-5p | 0.03 | 0.02 | 0.42 | 0.01 | 0.02 | 0.03 | 0.02 | 0.05 | 0.18 | 0.19 | 0.03 |
| hsa-miR-625-3p | 0.03 | 0.02 | 0.24 | 0.06 | 0.02 | 0.05 | 0.05 | 0.03 | 0.09 | 0.34 | 0.06 |
| hsa-miR-130b-3p | 0.03 | 0.01 | 0.13 | 0.04 | 0.00 | 0.01 | 0.05 | 0.01 | 0.45 | 0.26 | 0.02 |
| hsa-miR-3928 | 0.03 | 0.03 | 0.22 | 0.06 | 0.03 | 0.13 | 0.04 | 0.04 | 0.25 | 0.14 | 0.03 |
| hsa-miR-495 | 0.03 | 0.00 | 0.00 | 0.10 | 0.01 | 0.66 | 0.04 | 0.09 | 0.04 | 0.00 | 0.02 |
| hsa-miR-92b-5p | 0.03 | 0.00 | 0.52 | 0.01 | 0.01 | 0.20 | 0.04 | 0.08 | 0.09 | 0.01 | 0.00 |
| hsa-miR-195-5p | 0.03 | 0.13 | 0.01 | 0.11 | 0.01 | 0.02 | 0.02 | 0.08 | 0.03 | 0.02 | 0.54 |
| hsa-miR-425-3p | 0.02 | 0.03 | 0.05 | 0.03 | 0.03 | 0.20 | 0.01 | 0.05 | 0.46 | 0.06 | 0.04 |
| hsa-miR-320a | 0.02 | 0.01 | 0.45 | 0.09 | 0.04 | 0.03 | 0.04 | 0.03 | 0.16 | 0.11 | 0.02 |
| hsa-miR-4792 | 0.02 | 0.07 | 0.01 | 0.00 | 0.00 | 0.00 | 0.00 | 0.00 | 0.00 | 0.00 | 0.89 |
| hsa-let-7b-5p | 0.02 | 0.06 | 0.09 | 0.06 | 0.10 | 0.13 | 0.26 | 0.17 | 0.04 | 0.02 | 0.05 |
| hsa-miR-29b-3p | 0.02 | 0.12 | 0.25 | 0.04 | 0.06 | 0.22 | 0.03 | 0.14 | 0.04 | 0.03 | 0.06 |
| hsa-miR-29c-5p | 0.02 | 0.09 | 0.00 | 0.07 | 0.15 | 0.33 | 0.08 | 0.16 | 0.02 | 0.04 | 0.04 |
| hsa-miR-221-3p | 0.02 | 0.01 | 0.29 | 0.02 | 0.07 | 0.18 | 0.03 | 0.06 | 0.16 | 0.12 | 0.04 |
| hsa-miR-503 | 0.02 | 0.01 | 0.27 | 0.11 | 0.07 | 0.01 | 0.01 | 0.06 | 0.40 | 0.04 | 0.01 |
| hsa-miR-93-5p | 0.02 | 0.09 | 0.04 | 0.04 | 0.01 | 0.03 | 0.02 | 0.07 | 0.25 | 0.26 | 0.16 |
| hsa-miR-335-5p | 0.02 | 0.85 | 0.00 | 0.03 | 0.00 | 0.03 | 0.02 | 0.02 | 0.01 | 0.01 | 0.01 |
| hsa-miR-378a-3p | 0.02 | 0.19 | 0.21 | 0.12 | 0.11 | 0.00 | 0.01 | 0.02 | 0.09 | 0.05 | 0.17 |
| hsa-miR-766-3p | 0.02 | 0.06 | 0.05 | 0.02 | 0.01 | 0.10 | 0.01 | 0.03 | 0.19 | 0.42 | 0.09 |
| hsa-miR-216b | 0.02 | 0.00 | 0.00 | 0.00 | 0.00 | 0.00 | 0.98 | 0.00 | 0.00 | 0.00 | 0.00 |
| hsa-miR-181d | 0.02 | 0.00 | 0.02 | 0.01 | 0.02 | 0.58 | 0.05 | 0.04 | 0.21 | 0.05 | 0.01 |
| hsa-miR-760 | 0.02 | 0.01 | 0.09 | 0.00 | 0.03 | 0.81 | 0.01 | 0.02 | 0.01 | 0.01 | 0.00 |
| hsa-miR-34b-5p | 0.02 | 0.08 | 0.05 | 0.02 | 0.03 | 0.22 | 0.04 | 0.38 | 0.04 | 0.06 | 0.07 |
| hsa-miR-126-5p | 0.02 | 0.72 | 0.00 | 0.04 | 0.02 | 0.01 | 0.01 | 0.03 | 0.00 | 0.00 | 0.16 |
| hsa-miR-15b-3p | 0.02 | 0.05 | 0.06 | 0.03 | 0.04 | 0.04 | 0.01 | 0.10 | 0.11 | 0.28 | 0.27 |
| hsa-miR-4492 | 0.02 | 0.01 | 0.01 | 0.09 | 0.03 | 0.02 | 0.21 | 0.41 | 0.05 | 0.01 | 0.13 |
| hsa-miR-887 | 0.02 | 0.08 | 0.07 | 0.02 | 0.12 | 0.41 | 0.04 | 0.16 | 0.00 | 0.01 | 0.07 |
| hsa-miR-505-3p | 0.02 | 0.20 | 0.03 | 0.14 | 0.02 | 0.02 | 0.01 | 0.06 | 0.19 | 0.09 | 0.21 |
| hsa-miR-99a-3p | 0.02 | 0.09 | 0.01 | 0.17 | 0.11 | 0.23 | 0.07 | 0.05 | 0.01 | 0.00 | 0.24 |
| hsa-miR-708-5p | 0.02 | 0.38 | 0.00 | 0.01 | 0.01 | 0.23 | 0.02 | 0.03 | 0.01 | 0.02 | 0.29 |
| hsa-miR-25-3p | 0.02 | 0.00 | 0.17 | 0.04 | 0.03 | 0.03 | 0.06 | 0.07 | 0.15 | 0.42 | 0.01 |
| hsa-miR-145-3p | 0.02 | 0.64 | 0.00 | 0.03 | 0.03 | 0.02 | 0.04 | 0.05 | 0.00 | 0.00 | 0.16 |
| hsa-miR-664-5p | 0.02 | 0.02 | 0.05 | 0.13 | 0.11 | 0.16 | 0.18 | 0.12 | 0.05 | 0.15 | 0.02 |
| hsa-miR-23a-3p | 0.02 | 0.08 | 0.09 | 0.06 | 0.04 | 0.01 | 0.02 | 0.06 | 0.11 | 0.01 | 0.50 |
| hsa-miR-485-5p | 0.02 | 0.00 | 0.00 | 0.04 | 0.01 | 0.83 | 0.03 | 0.03 | 0.02 | 0.00 | 0.01 |
| hsa-miR-29a-3p | 0.02 | 0.02 | 0.31 | 0.14 | 0.04 | 0.14 | 0.03 | 0.08 | 0.08 | 0.08 | 0.05 |
| hsa-miR-152 | 0.01 | 0.11 | 0.32 | 0.07 | 0.08 | 0.02 | 0.04 | 0.06 | 0.06 | 0.03 | 0.21 |
| hsa-miR-320d | 0.01 | 0.01 | 0.25 | 0.12 | 0.06 | 0.08 | 0.08 | 0.07 | 0.23 | 0.06 | 0.02 |
| hsa-miR-342-5p | 0.01 | 0.04 | 0.01 | 0.05 | 0.01 | 0.10 | 0.01 | 0.09 | 0.21 | 0.44 | 0.03 |
| hsa-miR-424-5p | 0.01 | 0.16 | 0.03 | 0.27 | 0.02 | 0.01 | 0.01 | 0.12 | 0.30 | 0.02 | 0.04 |
| hsa-let-7d-5p | 0.01 | 0.01 | 0.37 | 0.14 | 0.04 | 0.04 | 0.03 | 0.03 | 0.16 | 0.14 | 0.02 |
| hsa-miR-221-5p | 0.01 | 0.00 | 0.11 | 0.03 | 0.04 | 0.11 | 0.03 | 0.05 | 0.36 | 0.24 | 0.02 |
| hsa-miR-576-5p | 0.01 | 0.05 | 0.06 | 0.10 | 0.04 | 0.01 | 0.04 | 0.03 | 0.10 | 0.45 | 0.11 |
| hsa-miR-15b-5p | 0.01 | 0.02 | 0.04 | 0.02 | 0.00 | 0.00 | 0.00 | 0.03 | 0.44 | 0.34 | 0.09 |
| hsa-miR-181b-5p | 0.01 | 0.01 | 0.13 | 0.01 | 0.01 | 0.25 | 0.02 | 0.06 | 0.16 | 0.32 | 0.02 |
| hsa-miR-30b-3p | 0.01 | 0.02 | 0.08 | 0.32 | 0.13 | 0.06 | 0.08 | 0.06 | 0.04 | 0.18 | 0.02 |
| hsa-miR-4420 | 0.01 | 0.00 | 0.00 | 0.02 | 0.00 | 0.00 | 0.00 | 0.02 | 0.59 | 0.34 | 0.01 |
| hsa-miR-215 | 0.01 | 0.04 | 0.00 | 0.74 | 0.01 | 0.01 | 0.11 | 0.05 | 0.02 | 0.02 | 0.00 |
| hsa-miR-365a-5p | 0.01 | 0.02 | 0.66 | 0.14 | 0.02 | 0.01 | 0.03 | 0.02 | 0.05 | 0.02 | 0.02 |
| hsa-miR-490-5p | 0.01 | 0.01 | 0.02 | 0.41 | 0.28 | 0.21 | 0.04 | 0.01 | 0.01 | 0.00 | 0.01 |
| hsa-miR-146a-5p | 0.01 | 0.15 | 0.40 | 0.02 | 0.00 | 0.00 | 0.00 | 0.02 | 0.02 | 0.17 | 0.19 |
| hsa-miR-214-3p | 0.01 | 0.07 | 0.00 | 0.47 | 0.02 | 0.00 | 0.07 | 0.14 | 0.00 | 0.00 | 0.21 |
| hsa-let-7g-5p | 0.01 | 0.02 | 0.04 | 0.01 | 0.02 | 0.03 | 0.02 | 0.02 | 0.39 | 0.41 | 0.02 |
| hsa-miR-330-5p | 0.01 | 0.00 | 0.23 | 0.01 | 0.01 | 0.54 | 0.02 | 0.02 | 0.09 | 0.08 | 0.00 |
| hsa-miR-548e | 0.01 | 0.01 | 0.07 | 0.08 | 0.02 | 0.01 | 0.02 | 0.02 | 0.35 | 0.40 | 0.01 |
| hsa-miR-22-5p | 0.01 | 0.13 | 0.12 | 0.22 | 0.14 | 0.08 | 0.04 | 0.13 | 0.06 | 0.03 | 0.05 |
| hsa-miR-1306-3p | 0.01 | 0.01 | 0.08 | 0.05 | 0.20 | 0.20 | 0.04 | 0.05 | 0.24 | 0.12 | 0.00 |
| hsa-miR-4448 | 0.01 | 0.03 | 0.07 | 0.06 | 0.04 | 0.12 | 0.19 | 0.14 | 0.17 | 0.02 | 0.14 |
| hsa-miR-106b-5p | 0.01 | 0.06 | 0.06 | 0.01 | 0.01 | 0.01 | 0.01 | 0.03 | 0.37 | 0.24 | 0.21 |
| hsa-miR-424-3p | 0.01 | 0.00 | 0.18 | 0.32 | 0.05 | 0.01 | 0.01 | 0.07 | 0.31 | 0.04 | 0.01 |
| hsa-miR-3656 | 0.01 | 0.05 | 0.00 | 0.03 | 0.08 | 0.02 | 0.11 | 0.14 | 0.01 | 0.00 | 0.55 |
| hsa-miR-1246 | 0.01 | 0.00 | 0.36 | 0.02 | 0.06 | 0.00 | 0.06 | 0.10 | 0.15 | 0.09 | 0.14 |
| hsa-miR-218-5p | 0.01 | 0.44 | 0.00 | 0.00 | 0.00 | 0.08 | 0.01 | 0.05 | 0.00 | 0.00 | 0.40 |
| hsa-let-7c | 0.01 | 0.07 | 0.02 | 0.16 | 0.10 | 0.28 | 0.13 | 0.10 | 0.03 | 0.03 | 0.07 |
| hsa-miR-1277-3p | 0.01 | 0.07 | 0.09 | 0.09 | 0.04 | 0.11 | 0.05 | 0.07 | 0.28 | 0.17 | 0.02 |
| hsa-miR-18a-5p | 0.01 | 0.29 | 0.03 | 0.02 | 0.00 | 0.01 | 0.00 | 0.01 | 0.21 | 0.20 | 0.22 |
| hsa-miR-365a-3p | 0.01 | 0.06 | 0.14 | 0.05 | 0.13 | 0.06 | 0.06 | 0.05 | 0.00 | 0.05 | 0.39 |
| hsa-miR-4521 | 0.01 | 0.01 | 0.71 | 0.01 | 0.00 | 0.00 | 0.00 | 0.00 | 0.10 | 0.15 | 0.01 |
| hsa-miR-1255a | 0.01 | 0.01 | 0.21 | 0.01 | 0.00 | 0.00 | 0.01 | 0.00 | 0.39 | 0.32 | 0.04 |
| hsa-miR-940 | 0.01 | 0.01 | 0.09 | 0.03 | 0.02 | 0.48 | 0.03 | 0.15 | 0.11 | 0.05 | 0.01 |
| hsa-miR-31-5p | 0.01 | 0.00 | 0.63 | 0.01 | 0.00 | 0.17 | 0.08 | 0.05 | 0.03 | 0.01 | 0.01 |
| hsa-miR-130a-3p | 0.01 | 0.03 | 0.13 | 0.08 | 0.01 | 0.02 | 0.03 | 0.07 | 0.49 | 0.03 | 0.10 |
| hsa-miR-4532 | 0.01 | 0.13 | 0.00 | 0.00 | 0.02 | 0.00 | 0.05 | 0.09 | 0.00 | 0.00 | 0.70 |
| hsa-miR-1291 | 0.01 | 0.00 | 0.01 | 0.17 | 0.16 | 0.14 | 0.11 | 0.17 | 0.16 | 0.04 | 0.04 |
| hsa-miR-9-5p | 0.01 | 0.00 | 0.00 | 0.00 | 0.00 | 0.96 | 0.00 | 0.00 | 0.00 | 0.00 | 0.01 |
| hsa-miR-128 | 0.01 | 0.00 | 0.04 | 0.01 | 0.02 | 0.76 | 0.00 | 0.01 | 0.02 | 0.13 | 0.00 |
| hsa-miR-100-5p | 0.01 | 0.12 | 0.02 | 0.18 | 0.00 | 0.03 | 0.01 | 0.01 | 0.00 | 0.00 | 0.61 |
| hsa-miR-1271-5p | 0.01 | 0.02 | 0.06 | 0.02 | 0.12 | 0.13 | 0.02 | 0.03 | 0.20 | 0.36 | 0.04 |
| hsa-miR-193b-3p | 0.01 | 0.21 | 0.05 | 0.07 | 0.12 | 0.03 | 0.07 | 0.03 | 0.00 | 0.02 | 0.38 |
| hsa-miR-383 | 0.01 | 0.01 | 0.00 | 0.15 | 0.01 | 0.77 | 0.02 | 0.01 | 0.00 | 0.00 | 0.04 |
| hsa-miR-378f | 0.01 | 0.07 | 0.25 | 0.22 | 0.14 | 0.01 | 0.03 | 0.01 | 0.13 | 0.10 | 0.03 |
| hsa-miR-190a | 0.01 | 0.93 | 0.00 | 0.00 | 0.00 | 0.01 | 0.00 | 0.00 | 0.00 | 0.00 | 0.05 |
| hsa-miR-224-5p | 0.01 | 0.46 | 0.00 | 0.05 | 0.03 | 0.00 | 0.05 | 0.02 | 0.01 | 0.00 | 0.36 |
| hsa-miR-139-5p | 0.01 | 0.10 | 0.00 | 0.07 | 0.05 | 0.53 | 0.02 | 0.17 | 0.00 | 0.00 | 0.05 |
| hsa-miR-497-5p | 0.00 | 0.19 | 0.00 | 0.11 | 0.04 | 0.10 | 0.07 | 0.15 | 0.01 | 0.00 | 0.34 |
| hsa-miR-92a-1-5p | 0.00 | 0.00 | 0.53 | 0.05 | 0.01 | 0.00 | 0.01 | 0.01 | 0.14 | 0.23 | 0.00 |
| hsa-miR-378a-5p | 0.00 | 0.45 | 0.03 | 0.07 | 0.27 | 0.01 | 0.01 | 0.03 | 0.02 | 0.03 | 0.08 |
| hsa-miR-20a-5p | 0.00 | 0.14 | 0.01 | 0.01 | 0.00 | 0.00 | 0.00 | 0.01 | 0.15 | 0.13 | 0.54 |
| hsa-miR-17-5p | 0.00 | 0.08 | 0.02 | 0.03 | 0.00 | 0.00 | 0.00 | 0.02 | 0.41 | 0.21 | 0.21 |
| hsa-miR-126-3p | 0.00 | 0.66 | 0.00 | 0.00 | 0.00 | 0.00 | 0.00 | 0.00 | 0.00 | 0.00 | 0.33 |
| hsa-miR-155-5p | 0.00 | 0.05 | 0.01 | 0.01 | 0.00 | 0.00 | 0.01 | 0.04 | 0.12 | 0.69 | 0.06 |
| hsa-miR-504 | 0.00 | 0.05 | 0.01 | 0.08 | 0.04 | 0.65 | 0.03 | 0.09 | 0.01 | 0.01 | 0.03 |
| hsa-miR-99a-5p | 0.00 | 0.07 | 0.00 | 0.60 | 0.03 | 0.10 | 0.03 | 0.02 | 0.01 | 0.00 | 0.13 |
| hsa-miR-1255b-5p | 0.00 | 0.01 | 0.11 | 0.12 | 0.07 | 0.02 | 0.05 | 0.07 | 0.16 | 0.37 | 0.01 |
| hsa-miR-449c-5p | 0.00 | 0.01 | 0.06 | 0.02 | 0.01 | 0.13 | 0.03 | 0.58 | 0.09 | 0.06 | 0.00 |
| hsa-miR-1278 | 0.00 | 0.01 | 0.21 | 0.09 | 0.04 | 0.03 | 0.03 | 0.04 | 0.14 | 0.40 | 0.01 |
| hsa-miR-33a-5p | 0.00 | 0.05 | 0.09 | 0.06 | 0.02 | 0.24 | 0.04 | 0.04 | 0.32 | 0.10 | 0.02 |
| hsa-miR-150-5p | 0.00 | 0.28 | 0.00 | 0.02 | 0.00 | 0.00 | 0.00 | 0.02 | 0.08 | 0.35 | 0.24 |
| hsa-miR-208b | 0.00 | 0.00 | 0.00 | 0.01 | 0.96 | 0.01 | 0.01 | 0.00 | 0.00 | 0.00 | 0.00 |
| hsa-miR-1261 | 0.00 | 0.01 | 0.32 | 0.25 | 0.00 | 0.01 | 0.00 | 0.01 | 0.10 | 0.08 | 0.20 |
| hsa-miR-548j | 0.00 | 0.03 | 0.10 | 0.21 | 0.04 | 0.03 | 0.19 | 0.05 | 0.17 | 0.16 | 0.02 |
| hsa-miR-1827 | 0.00 | 0.01 | 0.10 | 0.03 | 0.06 | 0.06 | 0.06 | 0.05 | 0.17 | 0.45 | 0.00 |
| hsa-miR-320b | 0.00 | 0.00 | 0.54 | 0.11 | 0.05 | 0.04 | 0.04 | 0.03 | 0.14 | 0.04 | 0.00 |
| hsa-miR-219-5p | 0.00 | 0.00 | 0.00 | 0.00 | 0.00 | 0.97 | 0.01 | 0.00 | 0.00 | 0.00 | 0.00 |
| hsa-miR-514a-3p | 0.00 | 0.01 | 0.41 | 0.00 | 0.00 | 0.01 | 0.00 | 0.10 | 0.00 | 0.01 | 0.45 |
| hsa-miR-4634 | 0.00 | 0.09 | 0.00 | 0.00 | 0.00 | 0.00 | 0.01 | 0.02 | 0.00 | 0.00 | 0.86 |
| hsa-miR-935 | 0.00 | 0.01 | 0.00 | 0.00 | 0.01 | 0.95 | 0.00 | 0.00 | 0.00 | 0.00 | 0.01 |
| hsa-miR-17-3p | 0.00 | 0.01 | 0.06 | 0.03 | 0.01 | 0.01 | 0.02 | 0.03 | 0.67 | 0.15 | 0.02 |
| hsa-miR-145-5p | 0.00 | 0.18 | 0.00 | 0.06 | 0.09 | 0.03 | 0.09 | 0.23 | 0.01 | 0.00 | 0.30 |
| hsa-miR-193a-5p | 0.00 | 0.12 | 0.12 | 0.22 | 0.03 | 0.02 | 0.10 | 0.18 | 0.07 | 0.03 | 0.11 |
| hsa-miR-138-5p | 0.00 | 0.00 | 0.05 | 0.00 | 0.00 | 0.84 | 0.00 | 0.06 | 0.00 | 0.03 | 0.01 |
| hsa-miR-199a-5p | 0.00 | 0.03 | 0.01 | 0.40 | 0.05 | 0.00 | 0.13 | 0.14 | 0.09 | 0.00 | 0.14 |
| hsa-miR-199b-5p | 0.00 | 0.03 | 0.01 | 0.40 | 0.05 | 0.00 | 0.13 | 0.15 | 0.09 | 0.00 | 0.14 |
| hsa-miR-140-5p | 0.00 | 0.01 | 0.10 | 0.06 | 0.04 | 0.11 | 0.04 | 0.11 | 0.18 | 0.31 | 0.04 |
| hsa-miR-4510 | 0.00 | 0.04 | 0.04 | 0.19 | 0.05 | 0.21 | 0.11 | 0.07 | 0.09 | 0.18 | 0.01 |
| hsa-miR-23b-5p | 0.00 | 0.01 | 0.19 | 0.22 | 0.18 | 0.09 | 0.09 | 0.13 | 0.04 | 0.03 | 0.01 |
| hsa-miR-107 | 0.00 | 0.00 | 0.28 | 0.12 | 0.02 | 0.13 | 0.03 | 0.06 | 0.15 | 0.20 | 0.00 |
| hsa-miR-944 | 0.00 | 0.01 | 0.00 | 0.04 | 0.11 | 0.00 | 0.00 | 0.01 | 0.00 | 0.03 | 0.79 |
| hsa-miR-885-5p | 0.00 | 0.00 | 0.00 | 0.43 | 0.02 | 0.54 | 0.00 | 0.00 | 0.00 | 0.00 | 0.00 |
| hsa-miR-150-3p | 0.00 | 0.04 | 0.00 | 0.00 | 0.01 | 0.01 | 0.00 | 0.01 | 0.23 | 0.68 | 0.01 |
| hsa-miR-4429 | 0.00 | 0.01 | 0.50 | 0.10 | 0.02 | 0.02 | 0.02 | 0.01 | 0.23 | 0.09 | 0.00 |
| hsa-miR-584-5p | 0.00 | 0.02 | 0.36 | 0.00 | 0.03 | 0.50 | 0.01 | 0.03 | 0.04 | 0.00 | 0.01 |
| hsa-miR-106a-5p | 0.00 | 0.10 | 0.02 | 0.01 | 0.00 | 0.01 | 0.00 | 0.02 | 0.50 | 0.26 | 0.08 |
| hsa-miR-133a | 0.00 | 0.00 | 0.00 | 0.00 | 0.98 | 0.00 | 0.00 | 0.01 | 0.00 | 0.00 | 0.00 |
| hsa-miR-378e | 0.00 | 0.02 | 0.26 | 0.28 | 0.22 | 0.01 | 0.04 | 0.03 | 0.09 | 0.04 | 0.01 |
| hsa-miR-505-5p | 0.00 | 0.01 | 0.17 | 0.51 | 0.02 | 0.01 | 0.01 | 0.04 | 0.09 | 0.12 | 0.01 |
| hsa-miR-320c | 0.00 | 0.00 | 0.53 | 0.11 | 0.05 | 0.05 | 0.04 | 0.04 | 0.16 | 0.02 | 0.00 |
| hsa-miR-363-3p | 0.00 | 0.05 | 0.06 | 0.00 | 0.00 | 0.01 | 0.01 | 0.01 | 0.36 | 0.47 | 0.03 |
| hsa-miR-143-5p | 0.00 | 0.42 | 0.00 | 0.05 | 0.08 | 0.04 | 0.09 | 0.12 | 0.01 | 0.00 | 0.17 |
| hsa-miR-185-5p | 0.00 | 0.04 | 0.29 | 0.10 | 0.07 | 0.12 | 0.03 | 0.11 | 0.14 | 0.08 | 0.01 |
| hsa-miR-193b-5p | 0.00 | 0.04 | 0.18 | 0.20 | 0.24 | 0.07 | 0.16 | 0.02 | 0.00 | 0.07 | 0.02 |
| hsa-miR-20b-5p | 0.00 | 0.15 | 0.03 | 0.00 | 0.01 | 0.01 | 0.01 | 0.02 | 0.30 | 0.32 | 0.14 |
| hsa-miR-9-3p | 0.00 | 0.00 | 0.01 | 0.00 | 0.00 | 0.97 | 0.00 | 0.00 | 0.00 | 0.00 | 0.00 |
| hsa-miR-211-5p | 0.00 | 0.00 | 0.98 | 0.00 | 0.00 | 0.01 | 0.00 | 0.00 | 0.00 | 0.00 | 0.02 |
| hsa-miR-452-5p | 0.00 | 0.58 | 0.00 | 0.01 | 0.04 | 0.00 | 0.04 | 0.04 | 0.00 | 0.00 | 0.29 |
| hsa-miR-223-5p | 0.00 | 0.01 | 0.00 | 0.01 | 0.00 | 0.00 | 0.00 | 0.02 | 0.86 | 0.09 | 0.00 |
| hsa-miR-509-3-5p | 0.00 | 0.00 | 0.92 | 0.00 | 0.00 | 0.00 | 0.00 | 0.05 | 0.00 | 0.00 | 0.01 |
| hsa-miR-378b | 0.00 | 0.01 | 0.52 | 0.22 | 0.05 | 0.01 | 0.01 | 0.01 | 0.10 | 0.07 | 0.01 |
| hsa-miR-203 | 0.00 | 0.00 | 0.00 | 0.00 | 0.00 | 0.00 | 0.00 | 0.00 | 0.00 | 0.00 | 1.00 |
| hsa-miR-509-5p | 0.00 | 0.00 | 0.93 | 0.00 | 0.00 | 0.00 | 0.00 | 0.04 | 0.00 | 0.00 | 0.01 |
| hsa-miR-378c | 0.00 | 0.02 | 0.36 | 0.18 | 0.20 | 0.01 | 0.02 | 0.03 | 0.12 | 0.05 | 0.01 |
| hsa-miR-25-5p | 0.00 | 0.00 | 0.44 | 0.02 | 0.00 | 0.01 | 0.01 | 0.01 | 0.25 | 0.27 | 0.00 |
| hsa-miR-142-5p | 0.00 | 0.49 | 0.00 | 0.01 | 0.00 | 0.00 | 0.00 | 0.03 | 0.12 | 0.16 | 0.19 |
| hsa-miR-144-5p | 0.00 | 0.49 | 0.00 | 0.01 | 0.01 | 0.00 | 0.01 | 0.03 | 0.00 | 0.00 | 0.45 |
| hsa-miR-144-3p | 0.00 | 0.83 | 0.00 | 0.00 | 0.01 | 0.00 | 0.01 | 0.05 | 0.03 | 0.00 | 0.05 |
| hsa-miR-2964a-5p | 0.00 | 0.00 | 0.00 | 0.00 | 0.00 | 0.98 | 0.00 | 0.00 | 0.00 | 0.00 | 0.00 |
| hsa-miR-205-5p | 0.00 | 0.00 | 0.00 | 0.00 | 0.00 | 0.00 | 0.00 | 0.00 | 0.00 | 0.00 | 1.00 |
| hsa-miR-499a-5p | 0.00 | 0.00 | 0.00 | 0.01 | 0.96 | 0.01 | 0.00 | 0.01 | 0.00 | 0.00 | 0.00 |
| hsa-miR-223-3p | 0.00 | 0.06 | 0.00 | 0.01 | 0.00 | 0.01 | 0.01 | 0.06 | 0.77 | 0.04 | 0.04 |
| hsa-miR-196a-5p | 0.00 | 0.29 | 0.02 | 0.00 | 0.01 | 0.00 | 0.00 | 0.03 | 0.00 | 0.00 | 0.64 |
| hsa-miR-133b | 0.00 | 0.00 | 0.00 | 0.00 | 0.98 | 0.00 | 0.00 | 0.01 | 0.00 | 0.00 | 0.00 |
| hsa-miR-196b-5p | 0.00 | 0.36 | 0.01 | 0.00 | 0.00 | 0.00 | 0.00 | 0.04 | 0.09 | 0.02 | 0.48 |
| hsa-miR-508-5p | 0.00 | 0.00 | 0.91 | 0.00 | 0.00 | 0.00 | 0.00 | 0.04 | 0.00 | 0.00 | 0.04 |
| hsa-miR-124-3p | 0.00 | 0.00 | 0.00 | 0.00 | 0.00 | 0.99 | 0.00 | 0.01 | 0.00 | 0.00 | 0.00 |
| hsa-miR-378d | 0.00 | 0.00 | 0.41 | 0.15 | 0.26 | 0.01 | 0.02 | 0.03 | 0.08 | 0.05 | 0.00 |
| hsa-miR-451a | 0.00 | 0.36 | 0.00 | 0.02 | 0.02 | 0.00 | 0.02 | 0.14 | 0.01 | 0.00 | 0.43 |
| hsa-miR-206 | 0.00 | 0.00 | 0.00 | 0.00 | 0.96 | 0.00 | 0.01 | 0.03 | 0.00 | 0.00 | 0.00 |
| hsa-miR-1 | 0.00 | 0.00 | 0.00 | 0.00 | 0.99 | 0.00 | 0.00 | 0.00 | 0.00 | 0.00 | 0.00 |
| hsa-miR-3591-5p | 0.00 | 0.00 | 0.00 | 1.00 | 0.00 | 0.00 | 0.00 | 0.00 | 0.00 | 0.00 | 0.00 |
| hsa-miR-122-5p | 0.00 | 0.00 | 0.00 | 1.00 | 0.00 | 0.00 | 0.00 | 0.00 | 0.00 | 0.00 | 0.00 |
| hsa-miR-20b-3p | 0.00 | 0.01 | 0.23 | 0.01 | 0.00 | 0.03 | 0.01 | 0.02 | 0.31 | 0.38 | 0.00 |
| hsa-miR-363-5p | 0.00 | 0.01 | 0.29 | 0.01 | 0.00 | 0.02 | 0.01 | 0.05 | 0.18 | 0.44 | 0.00 |
| hsa-miR-122-3p | 0.00 | 0.00 | 0.00 | 0.98 | 0.02 | 0.00 | 0.00 | 0.00 | 0.00 | 0.00 | 0.00 |
