# Supplementary material for: The miRNA Profile of Human Pancreatic Islets and Beta-Cells and Relationship to Type 2 Diabetes Pathogenesis
Source: PLoS One. 2013 Jan 25;8(1):e55272. doi: 10.1371/journal.pone.0055272 (PMC3555946; doi:10.1371/journal.pone.0055272)
Supplement: Table S4 — Results for the significantly enriched predicted target gene sets for islet-expressed miRNAs from the MAGENTA analysis with the 75 percentile cut-off used to determine significance. (DOCX) [file pone.0055272.s004.docx]

| **Prediction type** | **miRNA target-gene set** | **Effective genes in set** | **Significant genes expected** | **Significant genes observed** | **Enrichment value** | **Enrichment p-value** | **Enrichment q-value** |
| --- | --- | --- | --- | --- | --- | --- | --- |
| MiRanda | hsa-miR-424-5p | 1160 | 290 | 344 | 1.19 | 9.90E-06 | 2.82E-03 |
| MiRanda | hsa-miR-23a-3p | 1425 | 356 | 413 | 1.16 | 1.00E-05 | 2.82E-03 |
| MiRanda | hsa-miR-23b-3p | 1436 | 359 | 410 | 1.14 | 9.00E-05 | 2.82E-03 |
| MiRanda | hsa-miR-224-5p | 1329 | 332 | 381 | 1.15 | 1.40E-04 | 2.82E-03 |
| MiRanda | hsa-miR-376c-3p | 1351 | 338 | 384 | 1.14 | 1.40E-04 | 2.82E-03 |
| MiRanda | hsa-miR-10b-5p | 791 | 198 | 237 | 1.20 | 3.30E-04 | 2.82E-03 |
| MiRanda | hsa-miR-143-3p | 1204 | 301 | 343 | 1.14 | 4.40E-04 | 2.82E-03 |
| MiRanda | hsa-miR-214-3p | 1204 | 301 | 344 | 1.14 | 4.60E-04 | 2.82E-03 |
| MiRanda | hsa-miR-149-5p | 1003 | 251 | 291 | 1.16 | 5.20E-04 | 2.82E-03 |
| MiRanda | hsa-miR-181a-5p | 1474 | 369 | 411 | 1.11 | 6.00E-04 | 2.82E-03 |
| MiRanda | hsa-miR-22-3p | 764 | 191 | 227 | 1.19 | 6.20E-04 | 2.82E-03 |
| MiRanda | hsa-miR-377-3p | 1495 | 374 | 416 | 1.11 | 7.30E-04 | 2.82E-03 |
| MiRanda | hsa-miR-185-5p | 1391 | 348 | 389 | 1.12 | 8.80E-04 | 2.82E-03 |
| MiRanda | hsa-miR-212-3p | 1279 | 320 | 359 | 1.12 | 9.20E-04 | 2.82E-03 |
| MiRanda | hsa-miR-329 | 1021 | 255 | 293 | 1.15 | 9.20E-04 | 2.82E-03 |
| MiRanda | hsa-miR-15b-5p | 1159 | 290 | 328 | 1.13 | 1.04E-03 | 2.82E-03 |
| MiRanda | hsa-miR-199b-5p | 1054 | 264 | 300 | 1.14 | 1.27E-03 | 2.82E-03 |
| MiRanda | hsa-miR-141-3p | 1473 | 368 | 407 | 1.11 | 1.37E-03 | 2.82E-03 |
| MiRanda | hsa-miR-330-5p | 961 | 240 | 276 | 1.15 | 1.49E-03 | 2.82E-03 |
| MiRanda | hsa-miR-10a-5p | 842 | 211 | 244 | 1.16 | 1.55E-03 | 2.82E-03 |
| MiRanda | hsa-miR-199a-5p | 994 | 249 | 283 | 1.14 | 1.68E-03 | 2.82E-03 |
| MiRanda | hsa-miR-98-5p | 691 | 173 | 203 | 1.17 | 2.05E-03 | 2.82E-03 |
| MiRanda | hsa-miR-132-3p | 1360 | 340 | 377 | 1.11 | 2.22E-03 | 2.82E-03 |
| MiRanda | hsa-let-7b-5p | 744 | 186 | 217 | 1.17 | 2.36E-03 | 2.82E-03 |
| MiRanda | hsa-miR-200a-3p | 1416 | 354 | 390 | 1.10 | 2.76E-03 | 2.82E-03 |
| MiRanda | hsa-miR-134 | 910 | 228 | 260 | 1.14 | 2.80E-03 | 2.82E-03 |
| MiRanda | hsa-miR-181b-5p | 1613 | 403 | 439 | 1.09 | 2.83E-03 | 2.82E-03 |
| MiRanda | hsa-miR-217 | 1202 | 301 | 335 | 1.11 | 2.98E-03 | 2.82E-03 |
| MiRanda | hsa-let-7c | 714 | 179 | 208 | 1.16 | 3.03E-03 | 2.82E-03 |
| MiRanda | hsa-miR-320c | 1363 | 341 | 375 | 1.10 | 3.71E-03 | 2.82E-03 |
| MiRanda | hsa-miR-16-5p | 1152 | 288 | 321 | 1.11 | 3.88E-03 | 2.82E-03 |
| MiRanda | hsa-miR-92a-3p | 856 | 214 | 244 | 1.14 | 3.88E-03 | 2.82E-03 |
| MiRanda | hsa-miR-320d | 1318 | 330 | 363 | 1.10 | 3.88E-03 | 2.82E-03 |
| MiRanda | hsa-let-7e-5p | 794 | 199 | 228 | 1.15 | 4.08E-03 | 2.82E-03 |
| MiRanda | hsa-let-7a-5p | 707 | 177 | 205 | 1.16 | 4.16E-03 | 2.82E-03 |
| MiRanda | hsa-miR-18a-5p | 989 | 247 | 278 | 1.13 | 4.49E-03 | 2.82E-03 |
| MiRanda | hsa-let-7d-5p | 699 | 175 | 202 | 1.15 | 4.56E-03 | 2.82E-03 |
| MiRanda | hsa-miR-320b | 1414 | 354 | 387 | 1.09 | 4.72E-03 | 2.82E-03 |
| MiRanda | hsa-miR-24-3p | 857 | 214 | 244 | 1.14 | 4.78E-03 | 2.82E-03 |
| MiRanda | hsa-miR-320a | 1414 | 354 | 386 | 1.09 | 5.38E-03 | 2.82E-03 |
| MiRanda | hsa-miR-150-5p | 1003 | 251 | 281 | 1.12 | 5.55E-03 | 2.82E-03 |
| MiRanda | hsa-miR-15a-5p | 1148 | 287 | 318 | 1.11 | 5.94E-03 | 2.82E-03 |
| MiRanda | hsa-miR-485-5p | 1254 | 314 | 345 | 1.10 | 6.05E-03 | 2.82E-03 |
| MiRanda | hsa-miR-195-5p | 1142 | 286 | 316 | 1.10 | 6.34E-03 | 2.82E-03 |
| MiRanda | hsa-miR-181c-5p | 1379 | 345 | 376 | 1.09 | 6.78E-03 | 2.82E-03 |
| MiRanda | hsa-miR-551b-3p | 153 | 38 | 52 | 1.37 | 6.79E-03 | 2.82E-03 |
| MiRanda | hsa-miR-154-5p | 994 | 249 | 277 | 1.11 | 7.42E-03 | 2.86E-03 |
| MiRanda | hsa-miR-92b-3p | 796 | 199 | 226 | 1.14 | 7.65E-03 | 2.86E-03 |
| MiRanda | hsa-miR-136-5p | 1489 | 372 | 403 | 1.08 | 7.90E-03 | 2.86E-03 |
| MiRanda | hsa-miR-497-5p | 1051 | 263 | 291 | 1.11 | 9.02E-03 | 3.01E-03 |
| MiRanda | hsa-miR-181d | 1729 | 432 | 462 | 1.07 | 9.34E-03 | 3.02E-03 |
| TargetScan | hsa-miR-26b-5p | 252 | 63 | 89 | 1.41 | 1.40E-04 | 2.34E-02 |
| TargetScan | hsa-miR-26a-5p | 254 | 64 | 88 | 1.38 | 2.80E-04 | 2.34E-02 |
| TargetScan | hsa-miR-24-3p | 281 | 70 | 92 | 1.31 | 1.63E-03 | 2.34E-02 |
| TargetScan | hsa-miR-454-3p | 184 | 46 | 63 | 1.37 | 2.86E-03 | 2.34E-02 |
| TargetScan | hsa-miR-874 | 145 | 36 | 51 | 1.42 | 3.58E-03 | 2.34E-02 |
| TargetScan | hsa-miR-455-5p | 79 | 20 | 30 | 1.50 | 6.95E-03 | 2.34E-02 |
| TargetScan | hsa-miR-194-5p | 141 | 35 | 48 | 1.37 | 9.09E-03 | 2.34E-02 |
| TargetScan | hsa-miR-134 | 94 | 24 | 34 | 1.42 | 9.68E-03 | 2.34E-02 |
| TargetScan | hsa-miR-130a-3p | 187 | 47 | 61 | 1.30 | 9.83E-03 | 2.34E-02 |
| MiRanda+TargetScan | hsa-miR-93-5p | 873 | 218 | 257 | 1.18 | 3.70E-04 | 9.19E-03 |
| MiRanda+TargetScan | hsa-miR-200b-3p | 780 | 195 | 232 | 1.19 | 4.80E-04 | 9.19E-03 |
| MiRanda+TargetScan | hsa-miR-200c-3p | 784 | 196 | 232 | 1.18 | 5.10E-04 | 9.19E-03 |
| MiRanda+TargetScan | hsa-miR-20a-5p | 864 | 216 | 253 | 1.17 | 6.40E-04 | 9.19E-03 |
| MiRanda+TargetScan | hsa-miR-429 | 783 | 196 | 232 | 1.18 | 7.00E-04 | 9.19E-03 |
| MiRanda+TargetScan | hsa-miR-101-3p | 533 | 133 | 164 | 1.23 | 7.00E-04 | 9.19E-03 |
| MiRanda+TargetScan | hsa-miR-106b-5p | 865 | 216 | 251 | 1.16 | 1.29E-03 | 9.19E-03 |
| MiRanda+TargetScan | hsa-miR-27a-3p | 723 | 181 | 213 | 1.18 | 1.44E-03 | 9.19E-03 |
| MiRanda+TargetScan | hsa-miR-17-5p | 867 | 217 | 250 | 1.15 | 1.93E-03 | 9.19E-03 |
| MiRanda+TargetScan | hsa-miR-27b-3p | 723 | 181 | 211 | 1.17 | 2.35E-03 | 9.19E-03 |
| MiRanda+TargetScan | hsa-miR-539-5p | 423 | 106 | 130 | 1.23 | 2.83E-03 | 9.19E-03 |
| MiRanda+TargetScan | hsa-miR-98-5p | 515 | 129 | 154 | 1.19 | 3.62E-03 | 9.19E-03 |
| MiRanda+TargetScan | hsa-let-7c | 522 | 131 | 156 | 1.19 | 3.65E-03 | 9.19E-03 |
| MiRanda+TargetScan | hsa-miR-301b | 651 | 163 | 190 | 1.17 | 3.95E-03 | 9.19E-03 |
| MiRanda+TargetScan | hsa-miR-543 | 519 | 130 | 155 | 1.19 | 4.03E-03 | 9.19E-03 |
| MiRanda+TargetScan | hsa-miR-340-5p | 957 | 239 | 270 | 1.13 | 4.43E-03 | 9.19E-03 |
| MiRanda+TargetScan | hsa-miR-590-5p | 209 | 52 | 69 | 1.33 | 5.07E-03 | 9.19E-03 |
| MiRanda+TargetScan | hsa-miR-219-5p | 213 | 53 | 70 | 1.32 | 5.28E-03 | 9.19E-03 |
| MiRanda+TargetScan | hsa-miR-130b-3p | 639 | 160 | 186 | 1.16 | 5.36E-03 | 9.19E-03 |
| MiRanda+TargetScan | hsa-let-7b-5p | 523 | 131 | 155 | 1.18 | 5.39E-03 | 9.19E-03 |
| MiRanda+TargetScan | hsa-miR-197-3p | 111 | 28 | 40 | 1.43 | 5.64E-03 | 9.19E-03 |
| MiRanda+TargetScan | hsa-miR-23a-3p | 794 | 199 | 226 | 1.14 | 6.16E-03 | 9.19E-03 |
| MiRanda+TargetScan | hsa-miR-301a-3p | 649 | 162 | 188 | 1.16 | 6.60E-03 | 9.19E-03 |
| MiRanda+TargetScan | hsa-miR-130a-3p | 639 | 160 | 185 | 1.16 | 7.00E-03 | 9.19E-03 |
| MiRanda+TargetScan | hsa-let-7a-5p | 519 | 130 | 153 | 1.18 | 7.21E-03 | 9.19E-03 |
| MiRanda+TargetScan | hsa-miR-34a-5p | 372 | 93 | 113 | 1.22 | 8.21E-03 | 9.19E-03 |
| MiRanda+TargetScan | hsa-miR-421 | 289 | 72 | 90 | 1.25 | 8.51E-03 | 9.19E-03 |
| MiRanda+TargetScan | hsa-let-7d-5p | 518 | 130 | 152 | 1.17 | 8.85E-03 | 9.19E-03 |
| MiRanda+TargetScan | hsa-miR-454-3p | 643 | 161 | 185 | 1.15 | 9.36E-03 | 9.19E-03 |
| MiRanda+TargetScan | hsa-miR-23b-3p | 792 | 198 | 224 | 1.13 | 9.88E-03 | 9.19E-03 |
| MiRanda+miRDB | hsa-miR-374a-5p | 749 | 187 | 216 | 1.16 | 4.09E-03 | 5.73E-02 |
| MiRanda+miRDB | hsa-miR-130b-3p | 378 | 95 | 115 | 1.21 | 6.70E-03 | 5.73E-02 |
| MiRanda+miRDB | hsa-miR-155-5p | 332 | 83 | 102 | 1.23 | 8.31E-03 | 5.73E-02 |
| MiRanda+miRDB | hsa-miR-129-5p | 652 | 163 | 187 | 1.15 | 9.55E-03 | 5.73E-02 |
| MiRanda+miRDB+TargetScan | hsa-miR-17-5p | 476 | 119 | 143 | 1.20 | 4.43E-03 | 8.55E-02 |
| MiRanda+miRDB+TargetScan | hsa-miR-93-5p | 465 | 116 | 140 | 1.21 | 4.54E-03 | 8.55E-02 |
| MiRanda+miRDB+TargetScan | hsa-miR-20a-5p | 476 | 119 | 141 | 1.18 | 8.65E-03 | 8.55E-02 |
